# Biophysical and Functional Characterization of Rhesus Macaque IgG Subclasses

**DOI:** 10.3389/fimmu.2016.00589

**Published:** 2016-12-13

**Authors:** Austin W. Boesch, Nana Yaw Osei-Owusu, Andrew R. Crowley, Thach H. Chu, Ying N. Chan, Joshua A. Weiner, Pranay Bharadwaj, Rufus Hards, Mark E. Adamo, Scott A. Gerber, Sarah L. Cocklin, Joern E. Schmitz, Adam R. Miles, Joshua W. Eckman, Aaron J. Belli, Keith A. Reimann, Margaret E. Ackerman

**Affiliations:** ^1^Thayer School of Engineering, Dartmouth College, Hanover, NH, USA; ^2^Molecular and Cellular Biology Program, Dartmouth College, Hanover, NH, USA; ^3^Department of Genetics and Biochemistry, Geisel School of Medicine, Hanover, NH, USA; ^4^Norris Cotton Cancer Center, Geisel School of Medicine, Lebanon, NH, USA; ^5^Center for Virology and Vaccine Research, Beth Israel Deaconess Medical Center, Harvard Medical School, Boston, MA, USA; ^6^Wasatch Microfluidics, Salt Lake City, UT, USA; ^7^Non-Human Primate Reagent Resource, MassBiologics of the University of Massachusetts Medical School, Boston, MA, USA

**Keywords:** IgG, rhesus, effector function, non-human primate, Fc receptor

## Abstract

Antibodies raised in Indian rhesus macaques [*Macaca mulatta* (MM)] in many preclinical vaccine studies are often evaluated *in vitro* for titer, antigen-recognition breadth, neutralization potency, and/or effector function, and *in vivo* for potential associations with protection. However, despite reliance on this key animal model in translation of promising candidate vaccines for evaluation in first in man studies, little is known about the properties of MM immunoglobulin G (IgG) subclasses and how they may compare to human IgG subclasses. Here, we evaluate the binding of MM IgG1, IgG2, IgG3, and IgG4 to human Fc gamma receptors (FcγR) and their ability to elicit the effector functions of human FcγR-bearing cells, and unlike in humans, find a notable absence of subclasses with dramatically silent Fc regions. Biophysical, *in vitro*, and *in vivo* characterization revealed MM IgG1 exhibited the greatest effector function activity followed by IgG2 and then IgG3/4. These findings in rhesus are in contrast with the canonical understanding that IgG1 and IgG3 dominate effector function in humans, indicating that subclass-switching profiles observed in rhesus studies may not strictly recapitulate those observed in human vaccine studies.

## Introduction

Studies in non-human primates (NHP) are often a key aspect of preclinical vaccine development. Antibodies raised in NHP in such studies are often evaluated *in vitro* for titer, antigen-recognition breadth, and neutralization potency or effector function, as well as *in vivo* for potential associations with protection. These studies are conducted in settings ranging from simian immunodeficiency virus (SIV) or simian/human immunodeficiency virus, to TB, yellow fever, dengue, and malaria, among many others, with a goal of evaluating and understanding vaccine-mediated protection (1). However, despite reliance on NHP in translation of promising candidate vaccines and the established significance of antibody interactions with Fc gamma receptors (FcγR) as key contributions to antibody activity *in vivo*, little is known about the properties of NHP immunoglobulin G (IgG) subclasses. The widespread use of NHP in preclinical studies aimed at evaluating the possible efficacy of candidate vaccines, and the accumulating evidence that Fc-dependent activities strongly contribute to antibody-mediated protection, encourages functional characterization of IgG subclasses in these crucial model organisms.

For example, Indian rhesus macaques [*Macaca mulatta* (MM)] have been used extensively for evaluating antibody-based correlates of vaccine-mediated protection, and in several studies, antibody effector functions have been implicated as critical components of protection against SIV infection (2–5). However, despite these associations with protective efficacy, little is known about the functional characteristics of MM IgG subclasses. Reliance on these animal models in evaluation of candidate vaccines dictates closer evaluation of NHP IgG immunobiology, as despite evolutionary proximity, there may be important immunological differences between species.

In humans, antibody responses associated with differential IgG subclass compositions across different pathogen or antigen types are well known, and in some cases protection or pathology has been found to associate with variation in subclass selection. While a number of studies have investigated IgG subclasses in NHP (6, 7), the majority of this effort has been directed at defining subclass sequences; despite small study cohort sizes, this work has suggested significant allotypic diversity in IgA (8, 9). Previous experimental work has begun to elucidate the landscape of antibody properties in NHP but has not converged to yield a consensus understanding of the functions of primate IgG subclasses. Perplexingly, in an early experimental study aimed at functional characterization of cynomolgus macaque IgG subclasses, investigators observed similar subclass activities as in human (10), while a more recent investigation found divergent activity profiles between human and macaque IgG subclasses (11).

Here, we seek to evaluate the functional activity of MM IgG subclasses with the aim of enabling comparison of immunoglobulin biology between species. Evaluating species-mismatched antibodies and receptors is also relevant to the widespread use of human effector cells to evaluate the potency of rhesus serum IgGs elicited in vaccine studies, in which effector functions such as antibody-dependent cellular cytotoxicity (ADCC) (2, 5, 12–15), virus inhibition (ADCVI) (12, 16, 17), or phagocytosis (ADCP) (5, 15, 18) activities are often determined. These *in vitro* data help inform the potential mechanistic contributions of effector function to *in vivo* observations of protection or viremic control during vaccination or passive transfer studies in the NHP model and enable insight into vaccine and therapeutic design for humans. As a practical matter, the use of rhesus peripheral blood mononuclear cells (PBMCs) in effector assays is avoided due to their high inherent natural killer (NK) lytic activity and capacity to kill SIV-infected target cells. As a result, despite the species mismatch, the use of human target cells and human PBMCs in assessing the functional quality of rhesus antibody responses is specifically advised (2, 12, 19).

The use of human cell lines or PBMCs has enabled simple, reliable, and highly sensitive assays to assess rhesus macaque IgG effector functions and has eased comparison to *in vivo* results or to the *in vitro* functional activity of human IgGs. For example, the human PBMC ADCC activity of vaccine-elicited, serum-derived rhesus IgG has correlated to reduced acute viremia after intravaginal (2) and intrarectal (13) challenge with pathogenic SIV *in vivo*. Both ADCC and ADCVI assays using human PBMCs with rhesus anti-SIV IgG have been used to assess Ab potency in passive transfer studies (12). Additionally, the use of the human monocytic THP-1 cell line has been used to differentiate the phagocytic potential of vaccine-elicited rhesus serum IgG (18). Most importantly, such species-mismatched functional evaluations of antibody activity have correlated with protection from viral infection in recent challenge studies (5, 15).

Despite the prevalence of leveraging human effector cells to analyze the functionality of rhesus IgGs in vaccine and passive transfer studies, there is little known about the biophysical characteristics of rhesus IgG interacting with human FcγR. Here, we characterize the ability of recombinant rhesus IgG1, IgG2, IgG3, and IgG4 to interact with human FcγR and to induce the effector functions of human FcγR-bearing innate immune cells in order to investigate differences and similarities between species in terms of IgG subclass biology. As interspecies and intraspecies differences in IgG sequence and function pose potential caveats to the translation of findings in these key animal models, better understanding of antibody immunobiology in primates may prove key to enhancing the design and evaluation of future vaccine candidates.

## Materials and Methods

### Construction, Expression, and Purification of IgG and FcγR

To construct reference MM IgG of different subclasses, total RNA was isolated from 10^6^ mouse hybridoma cells expressing anti-CD8α monoclonal antibody, M-T807 (20) using RNAqueous-4PCR kit (Ambion Inc., Austin, TX, USA). A cDNA library was produced using SMARTer™ RACE cDNA Amplification Kit (Clontech, Mountain View, CA, USA). DNA encoding the variable domains of the heavy (V_H_) and the light chain (V_L_) were amplified using Advantage 2 PCR Kits (Clontech) with IgG1H (5′-ACCAAC GCTGCAGGTGACGGTCTGAC-3′) and IgG1k (5′-CTAACACTCATTCCTGTTGAAGCTCTTGAC-3′), cloned into the pCR 2.1-TOPO vector, used to transform One Shot TOP10 Chemically Competent *E. coli* (Invitrogen), and sequences confirmed. Three different rhesus recombinant versions of M-T807 were developed by grafting the CDRs, as defined by Kabat et al. (21), using rhesus Ig germline variable region sequences (NCBI accession numbers XP_001096027^1^ and XP_001106090^2^) as templates. Key positions potentially contributing to antigen binding, CDR conformation, and heavy–light chain interactions were back-mutated to the mouse residues. DNA representing recombinant V_H_ and V_L_ were subcloned into expression vectors containing rhesus IgG1 heavy chain (C_H_) and rhesus kappa light chain (C_L_) constant regions cloned from a rhesus B-lymphoblastoid cell line by PCR amplification. Rhesus recombinant antibodies representing each H + L chain combination were transiently expressed at small scale in HEK293 cells by co-transfection of recombinant light and heavy chain expression vectors using Lipofectamine (Invitrogen) and purified by Protein A affinity chromatography (GE Health Care). The CDR-grafted H + L chain pair that yielded the highest relative affinity antibody measured using a CD8α-expressing cell-based ELISA assay was used for all further experiments. The CDR-grafted V_H_ segments were also subcloned into rhesus IgG2, IgG3, or IgG4 C_H_ expression vectors, using rhesus gamma chain sequences previously reported (9), to permit expression of rhesus recombinant antibody of different IgG subclasses but sharing identical variable regions.

For large-scale production of rhesus antibodies, recombinant heavy and light chain vectors were packaged in retroviral vectors and used to infect CHO cells using the GPEx™ expression platform (Catalent Pharma Solutions, Madison, WI, USA). A pool of transduced cells was grown in serum-free medium from which recombinant antibodies were purified by protein A affinity chromatography and formulated in phosphate buffer at pH 6.5–8.0, based on each antibody’s isoelectric point. Endotoxin levels were <1 EU/mg of antibody. Recombinant rhesus antibody samples used in binding assays were SEC purified until aggregated IgG was undetectable (i.e., less than one part per thousand), and the monomeric fraction was used for all studies with the exception of IgG samples used in deglycosylation experiments.

Pooled polyclonal rhesus IgG purified from serum was obtained from the NHP Reagent Resource, and IgG from rhesus serum from a set of 47 Indian-origin rhesus macaques was evaluated either following dilution or after depletion of other common serum proteins using Melon gel (Thermo Pierce). Human IgG1, IgG2, and IgG4 myeloma proteins were obtained from Athens Research Corporation, while IgG3 myeloma protein was obtained from Sigma-Aldrich. The purity of these commercial preparations was described as >90%. The human VRC01 mAb was subclass-switched into IgG2, IgG3, and IgG4 forms as follows. Human IgG subclass backbone plasmids were obtained from Invivogen (pFUSEss-CHIg-hG2, pFUSEss-CHIg-hG3, pFUSEss-CHIg-hG4). The entire VRC-01 variable region was cloned out of the parent pCMV VRC01 IgG1 HC plasmid (NIH AIDS Reagent Program) and inserted into each individual subclass backbone plasmid. Sequencing was performed to confirm variable region insertion. Subsequently, each VRC-01 subclass gene was cloned back into an empty pCMV plasmid to maximize dual expression of the heavy chain with the pCMV VRC01 IgG1 LC plasmid (NIH AIDS Reagent Program).

The glycosylation profile of recombinant MM IgG subclasses and of polyclonal, serum-derived MM IgG was determined by HPLC as previously described (22). Human FcγRs were expressed and purified as previously described (23).

### MM Anti-Subclass Detection Reagents

A set of anti-MM IgG subclass detection monoclonal antibodies was developed as follows. Mice were immunized with baboon (*P. anubis*) serum IgG, and resulting hybridomas were screened for species and subclass specificity, resulting in isolation of a clone (7H11) with good specificity for MM IgG1, and minor recognition of MM IgG3. In order to generate monoclonal antibodies specific for MM IgG2 and IgG3, mice were immunized with peptides derived from the IgG hinge region, where MM subclasses demonstrate significant differences. The overlapping peptides VVHEPSNTKVDKTVGLPCRSTCPPCP and DKTVGLPCRSTCPPCPAELLGCPSVF were used to generate anti-IgG2 responses, whereas the peptide DKRVEFTPPCGDTTPPCPPPCPP was used to generate anti-IgG3 responses. Resulting antibodies were extensively screened for specificity and sensitivity, and clones 3C10 (anti-MM IgG2) and 2G11 (anti-MM IgG3) were identified. Similar approaches to immunize mice with IgG4 hinge peptides failed to generate monoclonal antibodies that bound specifically to MM IgG4. All reference IgGs and anti-subclass detecting antibodies are available through the NHP Reagent Resource.

### Structure Visualization and Manipulation

Immunoglobulin G alignments were generated using Geneious and ClustalW2. Sequences and coordinates for human IgG1 in complex with FcγRIIIb were retrieved from the Protein Data Bank (PDB) entry 1T89. FcγRI (PDB 4W4O) was aligned to FcγRIIIb (PDB 1T89), and contact residues were determined from the combined model using a cutoff distance of 5 Å. All structure manipulations and visualizations were performed in Chimera version 1.10.1 (24). Macaque sequences were retrieved from Scinicariello et al. (9), and human IgG sequences were from Uniprot P01857, P01859, P01860, and P01861. ClustalW2 alignments were used in Chimera to render by conservation. All protein structures shown are from 1T89 only.

### EndoS and PNGase F Treatment of IgG

For EndoS treatment of IgGs, 100 µg of MM monoclonal IgGs (NHP Reagent Resource) were treated with 2 µg of 1 mg/mL EndoS (gift from Dr. Kavitha Baruah from Oxford University) overnight at 37°C. EndoS is an enzyme from *S. pyogenes* that is highly specific for cleaving the IgG Fc glycan following the initial variably fucosylated *N*-acetylglucosamine (25). EndoS in the samples were inactivated by incubation at 55°C for 10 min. For PNGase F treatment of IgGs (to remove the entire Fc glycan), 0.5 µL of Remove-iT PNGase F (NEB) was added to each 100 µg sample of IgG and incubated for 24 h at 37°C. The PNGase F was then removed using chitin magnetic beads according to the protocol provided by New England Biolabs. Glycan removal was confirmed by running 2 µg of each DTT reduced IgG sample on a 4–12% bis-tris gel and observing a 3-kDa decrease in the molecular weight for the IgG heavy chain. Further confirmation was provided by lectin western blot (data not shown).

### Multiplex IgG Subclassing and Human FcγR Binding Assay

Purified, monomeric Hu FcγR or subclass-specific MM IgG detection reagents (7H11, 3C10, 2G11 clones, NHP Reagent Resource) were coupled to carboxylated magnetic beads (Luminex Corp.) using amine reactive chemistry as described previously (26). In an adaptation of a previously described protocol (23), antibody samples were serially diluted in 0.05% Tween-PBS in non-binding, clear-bottom black 384 well plates (Corning) in a total volume of 40 µL and for every dilution series, an additional well lacking test antibody was evaluated to determine background signal. A master bead mix containing all the receptor detection bead sets of interest was prepared in 0.05% Tween-PBS at a concentration of 50,000 beads/mL for each bead set. A 10-µL volume of this master bead mix was added to each well-containing antibody sample to reach a final volume of 50 µL. The plate was sonicated for 10–20 s to mix and then placed on an XYZ plate shaker (IKA) at 800 rpm for 2 h at room temperature. Unbound antibody was removed by washing the plate using an automatic plate washer (Biotek) fitted with a plate magnet and programed to conduct five washes with 60 µL of 0.05% Tween-PBS. Next, 40 µL of a 0.65 µg/mL goat anti-rhesus heavy and light chain-PE (Southern Biotech) in 0.05% Tween-PBS solution was added to each well, and the plate was sonicated and shaken at 800 rpm at room temperature for 1 h. The plate was washed again, and 35 µL of sheath fluid was used to resuspend beads in each well for analysis on a FlexMap 3D (Luminex Corp.). At least 35 beads for each bead set were analyzed. The average background MFI signal from wells that lacked antibody was subtracted from the signal from antibody-containing wells to account for any non-specific binding of detection reagent to beads. For all detection reagents, the magnitude of fluorescence observed from non-specific binding of detection reagent to the beads was comparable to that when beads are run in the absence of detection reagent (data not shown). Background-subtracted data were analyzed in GraphPad Prism, and non-linear regression trend lines were fit using the one site, specific binding model.

### ELISA Subclassing Assays

Specificity of MM anti-subclass detection antibodies was evaluated by ELISA, in which plates were functionalized with the extracellular domain of rhesus macaque CD8α protein, incubated with the recombinant anti-CD8α MM IgGs, and detected by the anti-subclass detection reagents. ELISA plates were coated overnight at 4°C with 0.5 µg/mL goat anti-HIS tag monoclonal antibody diluted in carbonate–bicarbonate buffer, pH 9.0, then blocked with Superblock at room temperature for 45 min. Recombinantly expressed and purified rhesus CD8α-HIS (NHP Reagent Resource) was applied at 5 µg/mL in 2% BSA for 1 h. Monoclonal anti-rhesus CD8α antibodies (IgG1–4) were applied for 1 h at 10 µg/mL in 2% BSA. Bound MM IgG was detected using the anti-rhesus IgG subclassing reagents described above diluted to 20 µg/mL in 2% BSA over a fourfold serial dilution range. After incubation for 1 h, bound subclass detection reagents were detected using a 1:1,000 dilution of a goat anti-mouse IgG (H + L) HRP in 2% BSA for 1 h, which was visualized with peroxidase substrate after a 4-min incubation at room temperature prior to stopping and reading out the product formation at OD 450 nm.

The presence of each IgG subclass in a single, pooled MM serum sample was evaluated similarly, with the exception that each recombinant MM IgG subclass and a MM serum sample were adhered directly to ELISA plates, followed by incubation with anti-subclass detection reagents over a dilution curve prior to development and absorbance measurements, essentially as described. Data were analyzed in GraphPad Prism, and non-linear regression trend lines were fit using the one site, specific binding model.

### LC–MS/MS Analysis of IgG Subclasses

*Macaca mulatta* serum from Indian-origin rhesus macaques and individual and defined mixtures of recombinant IgG subclass standards were reduced and separated by SDS-PAGE. Briefly, 10 µL of reduced and alkylated sample, at an estimated (serum) or exact (recombinant) IgG concentration of 0.167 mg/mL, was loaded into individual wells of a 15-well NuPAGE 4–12% bis-tris gel (ThermoFisher) and run at 200 V for 45 min, followed by visualization with Coomassie blue stain, band excision of the IgG HC, and in-gel digestion per standard procedures with sequencing grade trypsin (Promega). Peptide digests were analyzed by online microcapillary LC–MS/MS using an EasyLC-1000 UPLC and Q Exactive Plus mass spectrometer platform. Peptides were resuspended in 5% methanol/1% formic acid and loaded on to a trap column [1 cm length, 100 µm inner diameter, ReproSil, C_18_ AQ 5 µm 120 Å pore (Dr. Maisch, Ammerbuch, Germany)], vented to waste *via* a micro-tee, and eluted across a fritless analytical resolving column (35 cm length, 100 µm inner diameter, ReproSil, C_18_ AQ 3 µm 120 Å pore) pulled in-house (Sutter P-2000, Sutter Instruments, San Francisco, CA, USA) with a 60-min gradient of 5–30% LC-MS buffer B (LC-MS buffer A: 0.0625% formic acid, 3% ACN; LC-MS buffer B: 0.0625% formic acid, 95% ACN). The Q Exactive Plus was set to perform an Orbitrap MS1 scan (*R* = 70 K; AGC target = 3e6) from 350 to 1,500 Th, followed by HCD MS2 spectra on the 10 most abundant precursor ions detected by Orbitrap scanning (*R* = 17.5 K; AGC target = 1e5; max ion time = 75 ms) before repeating the cycle. Precursor ions were isolated for HCD by quadrupole isolation at width = 0.8 Th and HCD fragmentation at 26 normalized collision energy. Charge state 2, 3, and 4 ions were selected for MS2. Precursor ions were added to a dynamic exclusion list ± 20 ppm for 20 s. Raw data were searched using COMET (release version 2014.01) in high resolution mode (27) against a FASTA database containing all four IgG subclasses with a precursor mass tolerance of ±1 Da and a fragment ion mass tolerance of 0.02 Da, and requiring fully tryptic peptides (K, R; not preceding P) with up to three miscleavages. Static modifications included carbamidomethylcysteine, and oxidized methionine was the variable modification. Searches were filtered using orthogonal measures including mass measurement accuracy (±3 ppm), Xcorr for charges from +2 through +4, and dCn targeting a <1% FDR at the peptide level. Quantification of LC–MS/MS spectra was performed using MassChroQ (28). Annotated spectra and details of the peptides observed for each sample are provided in Files S1 and S2 in Supplementary Material, respectively.

### Surface Plasmon Resonance (SPR) Affinity Measurements

Surface plasmon resonance was used to measure equilibrium binding affinities. A Continuous Flow Microspotter (CFM) (Wasatch Microfluidics) was used to print up to 96 individual regions of interests (ROI) on a single gold prism surface coated with carboxymethyldextran substrate (200 M Xantec Bioanalytics). CFM fluid paths were primed with 25 mM sodium acetate pH 5.0 + 0.01% Tween 20 prior to activation. The substrate of each ROI was activated for 5–7 min with 100 µL of 1.2 mM *N*-hydroxysulfosuccinimide (sNHS) (Pierce) and 0.3 mM 1-ethyl-3-[3 dimethlyaminopropyl]carbodiimide-HCl (EDC) (Pierce) in deionized water under flow at 45 µL/min. Antibodies prepared at 50, 25, 12.5, and 6.25 µg/mL in 25 mM sodium acetate pH 4.5–5.0 and printed on the activated ROI at the four resulting ligand (IgG) densities. A direct IgG coupling method was used since a suitable rhesus-specific capture reagent with a sufficiently slow off rate across all rhesus IgG subclass could not be identified. The image-based array reader (MX96, IBIS Technologies) was primed with 25 mM sodium acetate pH 5.0 + 0.01% Tween 20 and the prism loaded and quenched with 120 µL of 0.5 M ethanolamine (Sigma-Aldrich) prior to priming, conditioning, and analyte injections. FcγRs were diluted in running buffer (PBS + 0.01% BSA + Tween 20) and injected over an eight part, threefold serial dilution series, generally starting between 18 and 41 µM, and consisting of the following steps: 0.5-min baseline, 5-min association, 5-min dissociation, and 0.5-min baseline. The prism was regenerated with 0.5 min 100 mM glycine pH 3.0 and 0.5 min baseline between each analyte (FcγR) dilution tested. ROI signal was double referenced using signal from blank injections and signal from uncoupled interspots to account for non-specific binding. Data were processed in Scrubber 2 (Biologic Software Ltd.) by kinetic analysis applying global analysis to determine the equilibrium dissociation constant, *K*_D_. Steady-state analysis to calculate *K*_D_ yielded similar values. Genetically and enzymatically deglycosylated human IgG1 and a panel of mAbs and Fc point mutants were used as controls and recapitulated the expected patterns of FcγR recognition preferences (data not shown). Experiments were repeated 2 to 12 times with different conjugation densities and print pH conditions. Data from two separate experiments using different preparations of FcγRs, each with multiple print spots for each IgG sample are presented.

### *In Vitro* Functional Assays

#### Natural Killer Cell Degranulation

The ADCC potential of MM IgG subclasses was assessed by an adaptation of previously described assays (29, 30) in which NK cell degranulation is measured based on CD107a surface expression, a marker correlated with human NK cell cytokine release, cytotoxic activity (29), and ADCC, in the presence of human IgGs (31, 32) as well as rhesus macaque serum samples (14). The NK-92 human NK cell line (NantKwest, formerly Conkwest) was cultured in tissue culture flasks at 37°C, 5% CO_2_, with passaging every 3–4 days from 50 to 600,000 cell/mL using RPMI 1640 supplemented with l-glutamine, horse, and bovine sera, 100 U/mL IL-2 (AIDS Reagent Resource), and MEM non-essential amino acids. Sterile, 96-well polystyrene plates (Costar) were coated overnight at 4°C with a dilution series of each test IgG, diluted in PBS. Plates were washed and blocked using 1% BSA in PBS at room temperature. Each well received 2–10 × 10^3^ and 0.2 test volumes of anti-human CD107a detection reagent in a total volume of 200 µL. On each plate, two blocked wells received 2.5 μg/mL PMA and 0.5 µg/mL ionomycin as positive controls, while two blocked wells containing no antibody or cell stimulant served as negative controls. Following an 1-h incubation of the plates under 37°C, 5% CO_2_ conditions, Brefeldin A (final concentration of 10 µg/mL) and mononensin (GolgiStop, final concentration of 6 µg/mL) were added to each well and the plates incubated for another 3–5 h. After incubation, cells were washed with cold PBS, pelleted at 400 × *g* in a chilled (4°C) centrifuge, and resuspended in fixative prepared in PBS. Fixation of cells occurred during a 15-min, room temperature incubation that was shielded from light, and the reaction was subsequently quenched using PBS containing 5% serum protein. The quenching buffer was used for a final wash before resuspension in PBSF (PBS + 0.1% BSA). Data were acquired on a MACSQuant flow cytometer and analyzed using Flowjo V10 and GraphPad Prism. Non-linear regression trend lines were fit using the one site, specific binding model. ADCC assays were repeated five times, either in duplicate or triplicate.

#### Antibody-Dependent Cellular Phagocytosis

The phagocytic activity of human myeloma and MM IgG subclasses was assessed by adaptation of previously described phagocytosis assay (33). Briefly, 1 µm, yellow–green fluorescent FluoSpheres^®^ Carboxylate-Modified Microspheres (Life Technologies) were conjugated with rhesus CD8α extracellular domain protein (NHP Reagent Resource, NIH, MM IgG subclass apparent affinity ranged 1.2- to 1.4-fold across subclasses) for evaluation of MM subclasses, or anti-human IgG Fab (Jackson Immunoresearch, human IgG subclass apparent affinity ranged 1.2- to 2.5-fold across subclasses) for analysis of human myeloma-derived subclasses, and diluted to 800,000 beads per 50 µL in RPMI 1640 with l-glutamine (Corning), 10% FBS (Biowest), and 1× Penicillin/Streptomycin/Amphotericin (Corning). The THP-1 human monocyte cell line (gift from Dr. Brent Berwin) was used as the effector cell population, and cells were diluted to 20,000 cells per 150 µL RPMI. The assay was run in a 96-well tissue culture plate by pipetting 50 μL of beads, then 150 µL of cells, and lastly 50 µL of RPMI alone or antibody diluted in RPMI at 5× the final target concentration. The cells were incubated at 37°C at 5% CO_2_ for 4 h. After incubation, cells were fixed as described for the degranulation assay. Data were acquired on a MACSQuant flow cytometer and analyzed using Flowjo V10. A background-subtracted phagocytosis score was calculated by multiplying the percentage of bead positive cells by their MFI, followed by subtraction of the phagocytosis score observed in the absence of antibody. Data were analyzed in GraphPad Prism, and non-linear regression trend lines were fit using the one site, specific binding model. ADCP assays were repeated five times, either in duplicate or triplicate. Equivalent opsonization of beads with each subclass was experimentally confirmed.

### *In Vivo* Depletion Activity

#### Antibody Administration to Animals

Indian rhesus monkeys (MM) were administered a single 50 mg/kg dose of recombinant MM IgG anti-CD8α antibodies comprising the four MM IgG subclasses by intravenous injection. Blood samples were periodically drawn to assess the effect of each subclass on depletion of CD8+ lymphocytes. For all injections and blood sampling, animals were anesthetized with ketamine HCl. All animals were maintained in accordance with the guidelines of the Committee on Animals for the Harvard Medical School and the Guide for the Care and Use of Laboratory Animals (34).

#### Lymphocyte Immunophenotyping

Lymphocytes in peripheral blood were immunophenotyped using EDTA-anticoagulated blood specimens in a whole blood lysis technique. Fluorochrome-conjugated antibodies were incubated with 100 µL of whole blood or lymph node cell suspension for 20 min at room temperature. Antibodies used were anti-CD3 (SP34)-APC, anti-CD4-FITC (L200) (BD Bioscience), and anti-CD8-PE (DK25, Dako, Inc., Carpenteria, CA, USA). Erythrocytes were lysed using the Immunoprep Reagent System and a TQ-Prep Workstation (Beckman Coulter). To reduce the background level of staining, lysed samples were washed with 1.0 mL PBS, centrifuged for 3 min at 300 × *g* and fixed in PBS/1% formalin. Specimens were routinely analyzed for immunofluorescence on a BD FACSCalibur (BD Bioscience) using a manually determined scattergate to gate lymphocytes. Absolute lymphocyte counts on blood specimens were obtained using an ADVIA 120 Hematology Analyzer (Siemens Medical Solutions USA, Malvern, PA, USA).

## Results

### Sequence and Structural Differences among IgG Subclasses

**Figure 1:**
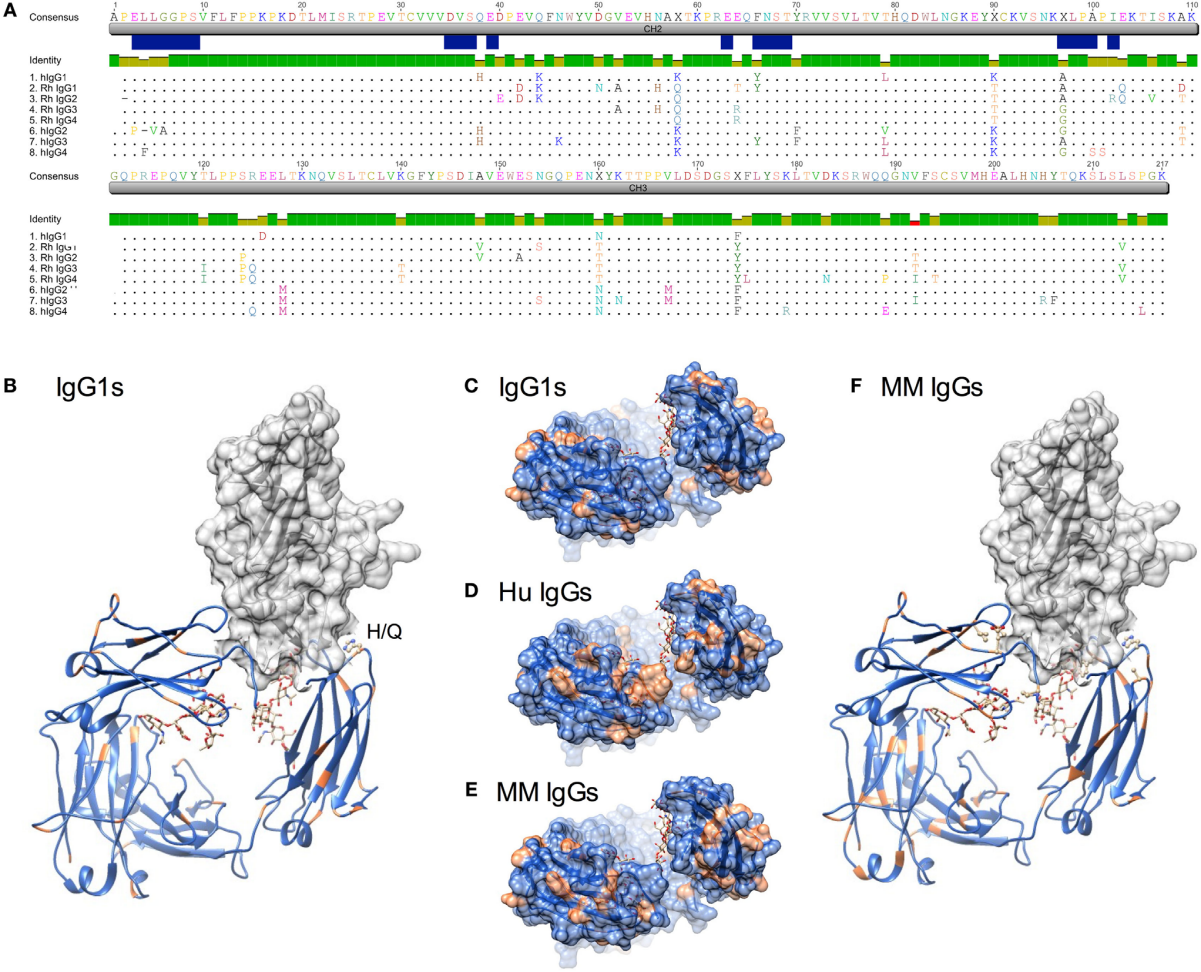
**Sequence and structural differences between human and *Macaca mulatta* (MM) immunoglobulin G (IgG)**. **(A)** Sequence alignment of human IgG subclasses with the recombinant monoclonal rhesus macaque IgG. CH2 and CH3 domains are denoted in gray, whereas the N-linked glycosylation site and known human IgG Fc gamma receptors (FcγR) contact residues (35–38) are annotated in blue. **(B)** Structural model of human IgG1 CH2 and CH3 domains (ribbon) in complex with human FcγRIIIa extracellular domain (space-fill) in which amino acids that differ between rhesus and human IgG1 sequences for each species are colored orange (pdb 1T89). H38Q and oligosaccharides at N297 are illustrated in ball and stick. **(C–E)**. A top-down view of the FcγR binding site, in which conserved positions are colored blue and variant positions colored orange. Oligosaccharides at N297 are illustrated in ball and stick. **(C)** Positional variance in the FcγR contact interface between human and MM IgG1. **(D)** Positional variance in the FcγR contact interface among the human IgG subclasses. **(E)** Positional variance in the FcγR contact interface among MM IgG subclasses. **(F)** Structural model of human IgG1 (ribbon) in complex with human FcγRIIIa (space-fill) in which amino acids that differ between MM IgG subclasses are colored orange. H38Q is illustrated in ball and stick.

The four IgG subclasses known to be present in MM were aligned with the CH2 domain of human IgG subclasses. Annotation of FcγR contact residues from co-crystal structures available for the high affinity FcγRI (35), low affinity FcγRIIa (36), and FcγRIIIa (37, 38) (Figure 1A) suggests that most MM IgG types are most similar to human IgG1 among predicted contact residues. As captured in a sequence identity matrix (Table 1), MM IgG were generally more similar to each other than to any human subclass. Thus, subclass numbering between species is not strictly consistent with overall sequence similarity between species across the entire Fc domain; further, rhesus subclasses tended to be more similar to human IgG1 than any other human subclass within the CH2 region responsible for recognition by FcγR. This sequence comparison suggests that MM IgG may function most similarly to human IgG1 and implies they may not exhibit the broad range of effector activities present among human IgG subclasses.

**Table 1:** **Relative Fc sequence conservation across species**.

	MM IgG1	MM IgG2	MM IgG3	MM IgG4
**CH1–hinge–CH2–CH3**				
Hu IgG1	90.7	88.5	89.2	88.8
Hu IgG2	87.1	88.3	87.7	88.1
Hu IgG3	78.5	76.4	78.2	76.4
Hu IgG4	87.4	87.2	89.2	90.2
MM IgG1	–	89.8	91	89.2
**CH2**				
Hu IgG1	90.0	89.1	90.9	92.7
Hu IgG2	82.7	84.5	88.2	90.0
Hu IgG3	87.3	87.3	89.1	90.9
Hu IgG4	85.5	86.4	91.8	93.6
MM IgG1	–	90.9	92.7	90.9

*Matrix presents the percent identity between the entire Fc and within the CH2 domain only*.

When sites of sequence variation between human and MM IgG1 are plotted on a rendering of the Fc–FcγR co-crystal structure, it is similarly apparent that most of the substitutions between species are located distal to FcγR contact residues (Figures 1B,C). One notable exception is H38, which borders an FcγR contact residue and is substituted to Q in all MM subclasses. In contrast, across the human IgG subclasses, a number of substitutions are present in or adjacent to contact residues (Figure 1A), particularly among those at the start of the CH2 domain near the hinge. Visualization of these sites of human subclass variation is consistent with their well-characterized differences in FcγR recognition (Figure 1D). In contrast, when MM subclasses are compared to each other, there are relatively fewer and generally more conservative substitutions at the interface (Figures 1E,F). However, several substitutions might be expected to impact FcγR recognition. First, MM IgG2 has a deletion of P2, proximal to FcγR contact residues. Second, MM IgG3 and IgG4 possess an A97G substitution in an FcγR contact residue. Interestingly, the A97G substitution is also present in human IgG2 and IgG4. Overall, structural assessment of sites of variation also suggests that MM subclass activities might be expected to vary less considerably than their human IgG counterparts.

### Biophysical Characterization of MM IgG Recognition by Human FcγR

**Figure 2:**
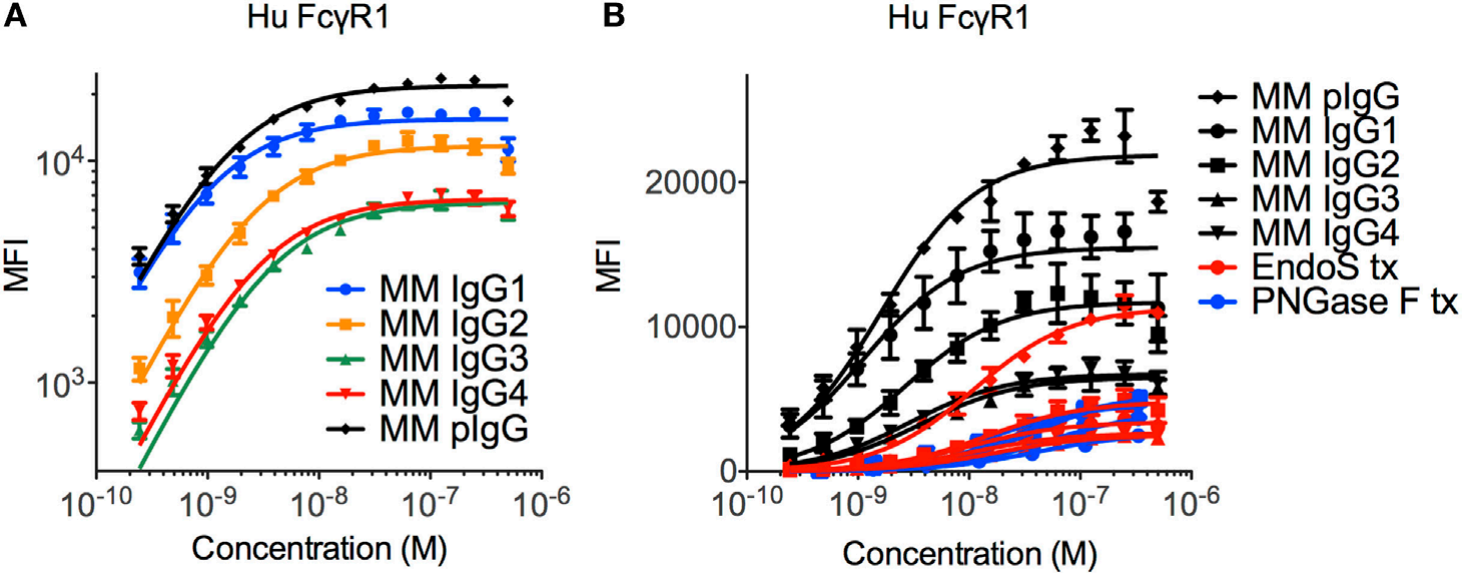
**Recognition of *Macaca mulatta* (MM) immunoglobulin G (IgG) by human FcγRI**. **(A)** Recombinant monoclonal rhesus macaque IgG (MM IgG1–4) with minor contamination with dimeric IgG and purified polyclonal rhesus macaque plasma IgG (MM pIgG) were titrated for binding to the high affinity human FcγRI. Triplicates representative of two independent experiments are presented. **(B)** IgG samples were treated with PNGaseF (blue) or EndoS (red) to remove either all or all but the variably fucosylated GlcNac core of the Fc domain glycan. Triplicate dilutions are presented.

The ability of the high affinity human FcγRI to recognize each MM subclass was determined in a multiplex assay that quantified IgG bound to FcγR-conjugated fluorescently coded microspheres. The high affinity human FcγRI receptor was observed to favor MM IgG1 > IgG2 > IgG3 and IgG4 (Figure 2A), in contrast to human IgGs, which are favored as IgG1/IgG3 > IgG4 > IgG2 (11, 39). The interaction between MM IgG and FcγRI was found to be highly dependent on IgG glycosylation (Figure 2B). Complete removal of N-linked IgG glycans with PNGase F ablated FcγRI recognition and glycan restriction with the IgG glycosidase EndoS, which leaves a core GlcNac that is variably fucosylated, likewise compromised binding.

**Figure 3:**
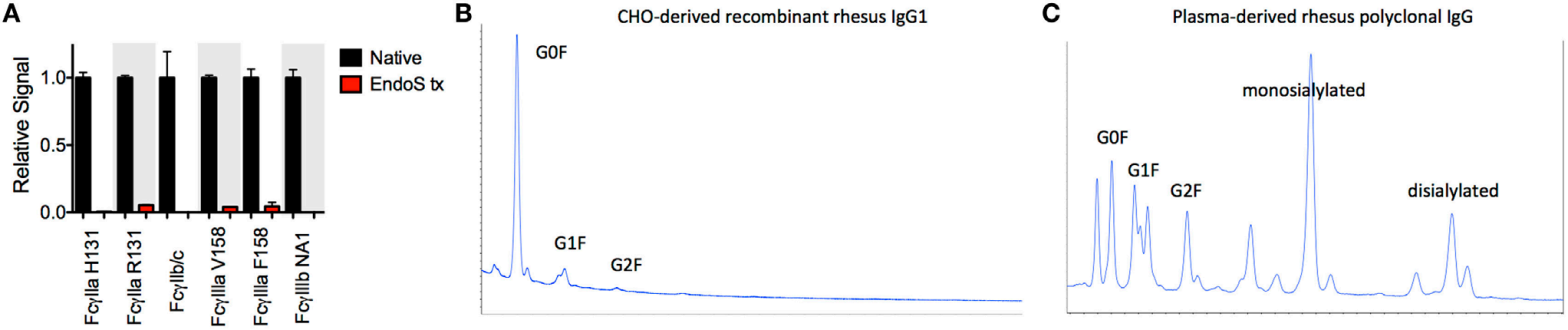
**Glycosylation state of *Macaca mulatta* (MM) immunoglobulin G (IgG) and glycan requirement for binding to low affinity human FcγR**. **(A)** Recombinant monoclonal rhesus macaque IgG (MM IgG1–4) with minor contamination with dimeric IgG and purified polyclonal rhesus macaque plasma IgG (MM pIgG) were either untreated or treated with EndoS (red) leaving a variably fucosylated GlcNac core of the Fc domain glycan. Triplicate measurements at 5 × 10^−7^ M MM IgG1 in one representative multiplex assay are presented. **(B,C)** IgG glycosylation as determined by glycan HPLC for MM IgG1 **(B)** and polyclonal IgG derived from MM serum **(C)**. Peak identities were determined by exoglycosidase digests and glycan controls.

Significant glycosylation sensitivity was also observed for the low affinity FcγRII and FcγRIII receptors using the multiplexing assay, where EndoS treatment significantly reduced the binding of MM IgG1 to FcγR-conjugated beads (Figure 3A). Glycan dependence was probed when IgG multimers were present in the antibody preparations to facilitate binding and indicated that glycan was necessary for recognition of even multimeric IgG. Similar results were observed following treatment with PNGaseF and across all MM subclasses and polyclonal serum IgG (data not shown). While the glycosylation profiles of CHO-derived MM IgG subclasses were relatively uniform and dominated by fucosylated non- and mono-galactosylated (G0F and G1F) species, serum-derived MM IgG exhibited a considerably more complex glycoprofile including many peaks comprised of sialylated glycoforms (Figures 3B,C).

**Figure 4:**
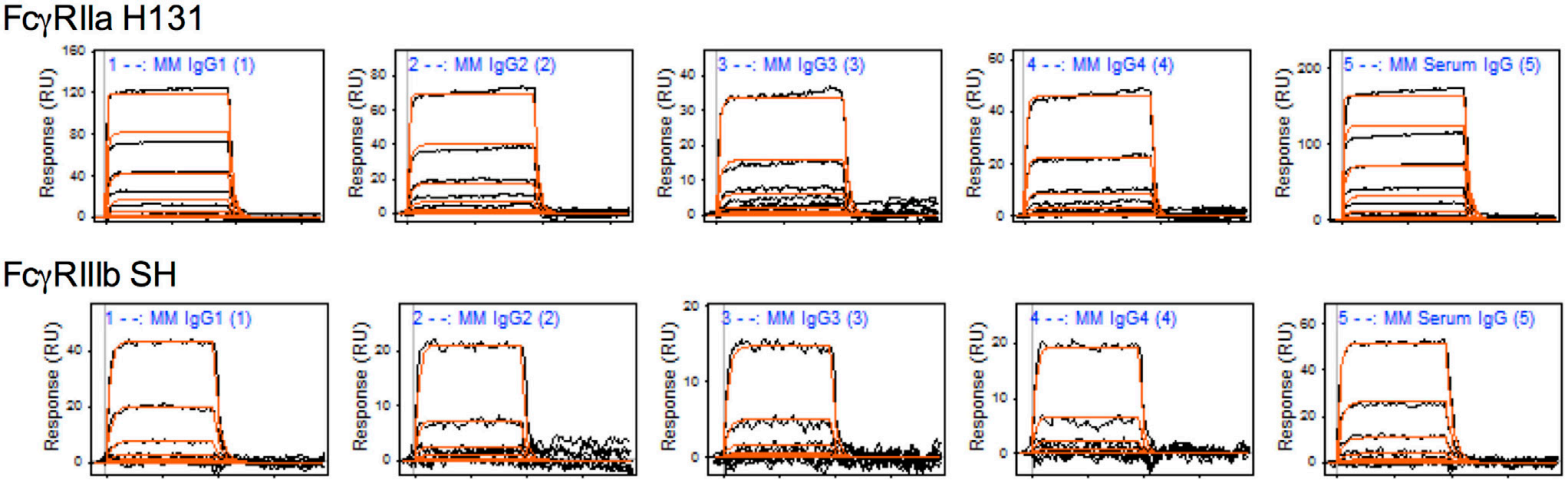
**Representative surface plasmon resonance (SPR) data and curve fits**. Raw SPR signals (black) and kinetic curve fits (red) in a 1:1 model are presented for the *Macaca mulatta* immunoglobulin G (IgG) subclasses and a purified serum IgG sample for FcγRIIa H131 and FcγRIIIb SH allotypes.

To characterize the affinity of monomeric MM IgG subclasses and rhesus serum-derived polyclonal IgG toward human FcγRII and FcγRIII receptors, a series of label-free kinetic experiments was conducted. Importantly, the use of SPR for this purpose offers greater sensitivity in characterizing low affinity interactions and enabled evaluation without reliance on detection reagents, which have the potential to introduce confounding factors. Indeed, the anti-CH1 reagents commonly employed to immobilize antibodies reliably maintained association to some but not all MM IgG subclasses, and therefore antibodies were covalently conjugated directly to SPR chips. The mean binding affinities (*K*_D_) for MM IgG subclasses across human FcγR allotypic variants were determined by global fitting of kinetic data using a 1:1 model (Table 2), and representative curve fits are shown in Figure 4. FcγRIII variants showed a strong preference toward MM IgG1 relative to all other subclasses, whereas FcγRII variants exhibited less variable affinities across MM subclasses. Notably, a sample of purified polyclonal serum IgG exhibited binding affinities with good agreement to MM IgG1.

**Table 2:** **Affinity of *Macaca mulatta* (MM) immunoglobulin G (IgG) subclasses for low affinity human FcγR**.

	FcγR	MM IgG1	MM IgG2	MM IgG3	MM IgG4
Experiment 1	FcγRIIa H131	7 ± 1	11 ± 4	27 ± 9	23 ± 5
	FcγRIIa R131	9 ± 4	32 ± 7	11 ± 2	11 ± 2
	FcγRIIb/c	36 ± 7	90 ± 43^a^	46 ± 12^a^	33 ± 3
	FcγRIIIa V158	4 ± 2	21 ± 13	84 ± 84^a^	32 ± 11^a^
	FcγRIIIa F158	4 ± 1	48 ± 51^a^	78 ± 52^a^	48 ± 26^a^
	FcγRIIIb NA1	18 ± 7	700 ± 430^a^	700 ± 430^a^	500 ± 370^a^
	FcγRIIIb NA2	13 ± 5	400 ± 420^a^	510 ± 370^a^	730 ± 380^a^
	FcγRIIIb SH	15 ± 8	530 ± 340^a^	740 ± 370^a^	465 ± 400^a^
Experiment 2	FcγRIIa H131	9 ± 2	11 ± 4	16 ± 4	20 ± 10
	FcγRIIa R131	21 ± 9	41 ± 16^a^	25 ± 20	20 ± 6
	FcγRIIb/c	53 ± 14^a^	125 ± 37^a^	38 ± 7	32 ± 7^a^
	FcγRIIIa V158	5 ± 2	14 ± 11	27 ± 33	29 ± 17
	FcγRIIIa F158	8 ± 2	15 ± 4	33 ± 4^a^	17 ± 1
	FcγRIIIb NA1	14 ± 6	250 ± 320^a^	210 ± 170^a^	120 ± 80^a^
	FcγRIIIb NA2	15 ± 3	32 ± 2^a^	270 ± 420^a^	270 ± 67^a^
	FcγRIIIb SH	9 ± 4	80 ± 100^a^	350 ± 860^a^	340 ± 500^a^

*Binding affinities (*K*_D_) of MM IgG subclasses for low affinity human FcγR allotypic variants as determined by surface plasmon resonance. Data are reported as the mean in micromolar ± SD across at least three independent measurements*.

*^a^Value is outside of the concentration range used and is therefore less reliable*.

### Composition and Properties of Polyclonal MM Serum IgG

**Figure 5:**
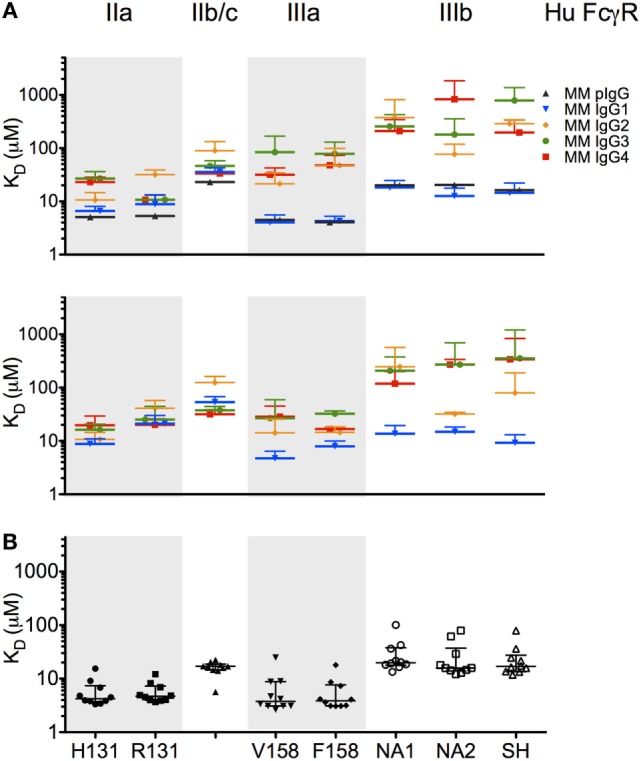
**Affinity of rhesus immunoglobulin G (IgG) for low affinity human FcγR**. **(A,B)** Equilibrium binding affinity (*K*_D_ in micromolar) of recombinant monoclonal rhesus IgG subclasses [*Macaca mulatta* (MM) IgG1–4] as well as a commercially supplied sample of purified polyclonal rhesus macaque plasma IgG (MM pIgG) to the low affinity human FcγR. Bars denote the mean of two to six replicates, in two separate experiments (upper and lower panels) using different preparations of FcγR. **(B)** Equilibrium binding affinity (*K*_D_ in micromolar) of 10 randomly selected purified rhesus macaque plasma IgG samples. Bars and whiskers denote the median and interquartile range.

To further evaluate whether the recombinantly produced MM IgGs reflected the behavior of naturally derived plasma IgG secreted by B cells, we conducted SPR experiments on a set of purified polyclonal antibodies from 10 MM (Figure 5B). Consistent with the commercially available preparation in Figure 5A, natural IgG exhibited an FcγR-binding profile highly consistent with MM IgG1, suggesting that MM serum IgG may be predominantly comprised of IgG1 or allotypic variants of other subclasses with IgG1-like FcγR binding. While IgG mixtures with different fractionation profiles have been resolved chromatographically (6), a general understanding of the subclass composition of MM plasma IgG has been lacking.

**Figure 6:**
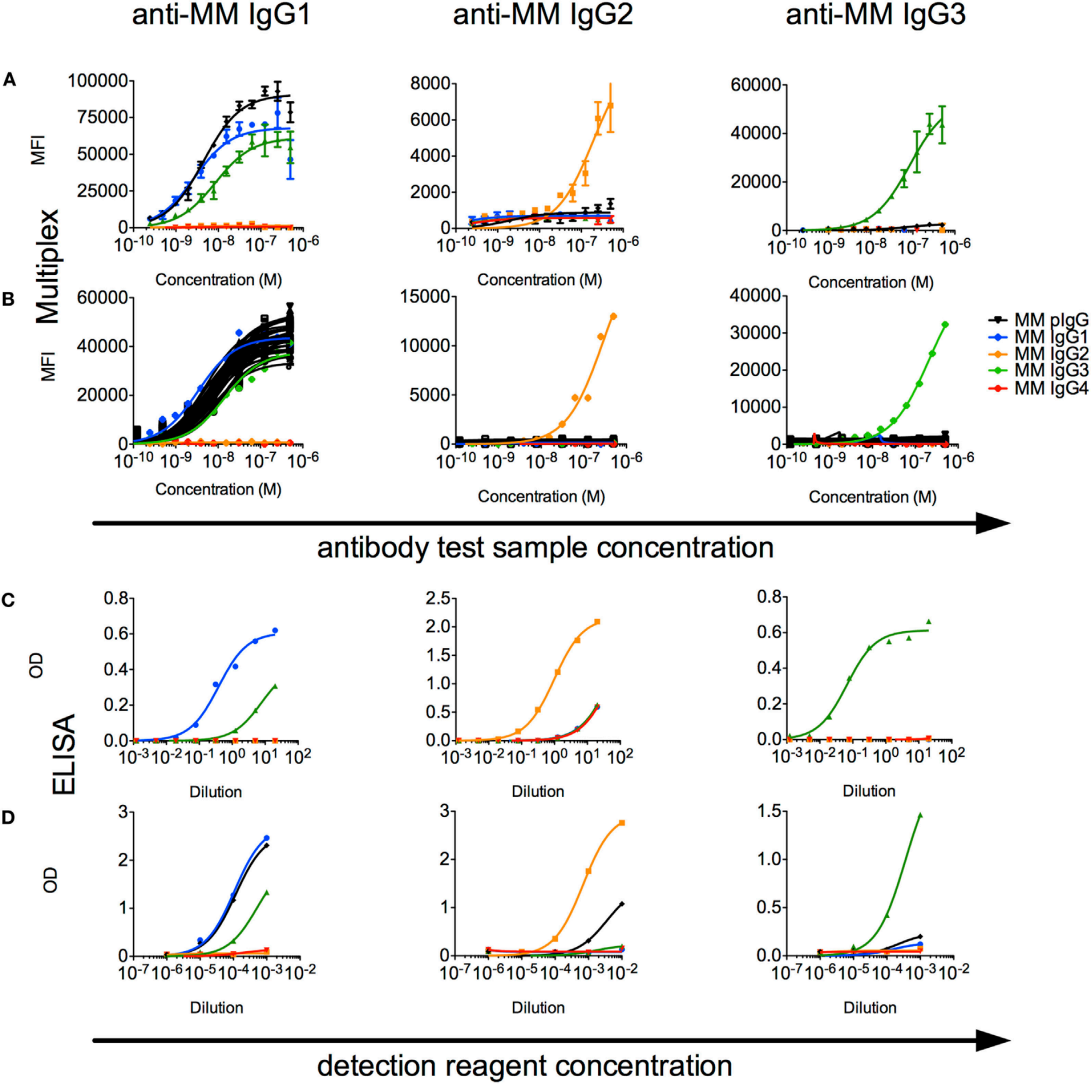
**Characterization of rhesus immunoglobulin G (IgG) subclass composition and detection reagents**. **(A,B)** Multiplex subclassing assays. **(A)** Recombinant monoclonal rhesus macaque IgG [*Macaca mulatta* (MM) IgG1–4] and pooled purified polyclonal rhesus macaque plasma IgG (MM pIgG) were titrated for binding to microspheres conjugated with anti-MM, subclass-specific antibodies (anti-MM IgG1, anti-MM IgG2, anti-MM IgG3), and bound test antibody detected by a pan anti-rhesus IgG detection antibody. **(B)** In a separate experiment, antibody purified from serum samples from 36 naïve MM (black) was likewise evaluated to detect the presence of IgG1, IgG2, and IgG3 subclasses. **(C,D)**. ELISA subclassing assays. **(C)** Sensitivity and specificity of MM subclass detection reagents over a titration range when recombinant, CD8α-specific MM IgG subclass antibody test samples were bound to CD8α-conjugated ELISA plates. **(D)** The composition of MM serum IgG in a pooled sample was evaluated *via* an ELISA in which either non-specifically adhered serum IgG or recombinant MM IgG subclasses as controls were detected using a dilution series of the anti-subclass detection reagents.

A limited number of studies report use of subclass-specific detection reagents (40). We evaluated a set of α-MM IgG subclass detection reagents for their subclass specificity and sensitivity. In a multiplex assay, we observed that IgG2 and IgG3 could be reliably distinguished from other recombinant subclasses using detection reagents raised against their hinges (Figure 6A), but that the available α-MM IgG1 detection antibody exhibited some cross-reactivity with MM IgG3. No detection reagent for MM IgG4 was available. Similar results were observed when these subclass detection antibodies were used in an ELISA format to detect recombinant CD8α-specific MM IgG bound to plates coated with CD8α (Figure 6C).

When these detection reagents were used to characterize the subclass composition of plasma IgG across a set of 36 MM serum IgG samples in a multiplexed fluorescent microsphere assay, only low levels of IgG2 or IgG3 were observed (Figure 6B). To understand the sensitivity limits of these reagents, analysis of defined mixtures of the recombinant monoclonals indicated that IgG2 and IgG3 could be detected even when prevalent at less than 10% by mass. When these reagents were used in ELISA format to detect MM subclasses from recombinant mAbs and polyclonal sera adhered to plates, IgG2 and IgG3 could be detected above background in the single polyclonal sera sample evaluated in this format (Figure 6D). Using absorbance signal to estimate concentration, however, indicated that IgG2 and IgG3 are likely minor components.

We next sought to assess IgG subclass concentrations in serum using semi-quantitative mass spectrometry. Manual inspection of the IgG subclass protein sequences suggested that subclass-unique tryptic peptide sequences could be used to both confirm the identity of individual subclasses within mixed samples or serum, as well as quantify the relative abundances in these samples. To do so, recombinant purified IgG subclasses were analyzed as pure samples as well as diluted in known ratios to one another to produce mixed IgG samples for development of a standard curve. These mixed standards were then separated by SDS-PAGE, digested with trypsin, and analyzed by high performance mass spectrometry. Unique peptide sequences were identified for all subclasses except IgG3; the peptide sequence that uniquely identifies this subclass is large and hydrophobic, which precluded its routine analysis from these mixtures. For the remaining three subclasses, peptide peak area standard curves were generated as a fraction of the total IgG analyzed, which enabled linear regression-based quantification of IgG subclasses 1, 2, and 4 from macaque sera (Table 3). Consistent with results noted above, IgG1 was the most abundant of the subclasses in these samples, followed by IgG2 and IgG4. Although IgG3 was unambiguously qualitatively identified in some of these serum samples, the poor quantitative performance of its unique peptide prohibited accurate estimation of its abundance.

**Table 3:** **Estimates of subclass prevalences in *Macaca mulatta* serum based on MS**.

	Animal																		
	1	2	3	4	5	6	7	8	9	10	11	12	13	14	15	16	17	18	19
IgG1	++++	+++++	++++	++++	+++	+++	++++	+++	+++++	++++	++++	++++	+++++	++++	++++	+++	++++	+++	++++
IgG2	+	++	+	+	++	+	+	+	++	+	++	++	++	++	++	++++	++	+++	++
IgG3	nd	nd	nd	nd	nd	nd	*	nd	*	nd	nd	nd	Nd	nd	*	nd	*	*	nd
IgG4	+	+	+	+	+	+	+	+	+	+	+	+	+	+	+	+	+	+	+

Response magnitudes are characterized as follows: nd, not observed; *, not quantified; +, <20%; ++, 20–40%; +++, 40–60%; ++++, 60–80%; +++++, 80–100%.

### Effector Function of MM IgG Subclasses

**Figure 7:**
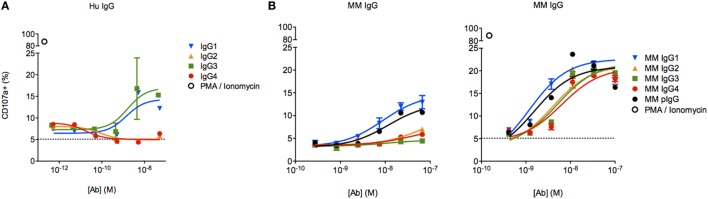
**Characterization of natural killer (NK) cell degranulation activity of *Macaca mulatta* (MM) immunoglobulin G (IgG) subclasses and comparison to human IgG subclasses**. **(A)** Subclass-switched forms of the human VRC01 mAb were evaluated in for ability to drive degranulation of NK-92 cells by directly coating a dilution series of IgG onto a 96-well plate. **(B)** Recombinant rhesus macaque IgG (MM1–4) and polyclonal rhesus macaque plasma IgG (pIgG) was likewise evaluated. Results from an assay with less responsive (left, *n* = 1) or normally responsive NK-92 cells (right, *n* = 4) are presented.

To assess the functional impact(s) of observed differences in FcγR binding between MM subclasses, we next evaluated the activity of each subclass in an assay measuring antibody-driven induction of human NK CD107a surface expression *via* cross-linking of FcγRIIIa V158, a surrogate indicator of NK cell cytokine release (30), cytotoxicity (29), and ADCC (14, 31, 32). Subclass-switched versions of a human mAb were able to induce NK activation only in IgG1 and IgG3 forms (Figure 7A). In contrast, all MM IgG subclasses were capable of activating the same preparation of NK-92 cells (Figure 7B), though one experiment with less responsive NK-92 cells more dramatically differentiated among MM subclasses. Consistent with biophysical data, this functional assessment demonstrated that MM IgG1 and serum-derived MM pIgG exhibited the greatest induction of NK cell activity.

**Figure 8:**
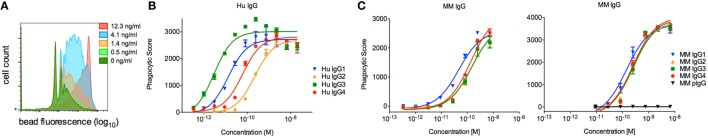
**Characterization of the phagocytic activity of *Macaca mulatta* (MM) immunoglobulin G (IgG) subclasses and comparison to human IgG subclasses**. **(A)** Exemplary flow cytometry histograms for a titration of MM IgG1 driving phagocytosis of CD8α-conjugated fluorescent beads. **(B)** Myeloma-derived human IgG1–4 were evaluated in the same assay measuring uptake of fluorescent beads, in this case using beads conjugated with an anti-human Fab reagent. Results from one representative experiment with technical replicates are presented. **(C)** Recombinant monoclonal rhesus macaque IgG (MM IgG1–4) was evaluated for its ability to drive phagocytosis of CD8α-conjugated fluorescent beads. Representative results from two of five independent experiments in which duplicate titrations were assessed are presented.

To evaluate the effector function of a phagocytic human effector cell with a more complex FcγR expression profile, we next evaluated the activity of each subclass in a commonly utilized assay of ADCP, in which monocytic human THP-1 cells are used as effectors and their uptake of antibody-opsonized fluorescent beads is quantified by flow cytometry (33). Beads were covalently conjugated with CD8α, and the phagocytic activity of each MM IgG subclass was determined across a titration range (Figure 8A). THP-1 cells express a range of FcγRs, including FcγRI, FcγRIIa, FcγRIIb, and sometimes FcγRIIIa, but phagocytic activity is most tightly linked to FcγRII ligation with some contribution from FcγRI (33, 41). In contrast to human IgG subclasses (Figure 8B), which exhibit dramatically different phagocytic potencies, MM IgGs demonstrated relatively consistent activity when either the Fv target antigen CD8α (Figure 8C) or an anti-MM IgG polyclonal antibody-based capture (data not shown) was used to form immune complexes. This ADCP activity profile observed was consistent with affinity for FcγRII by SPR. Differences in phagocytic activity among MM subclasses were considerably less pronounced than activity differences observed in the NK activation assay (Figure 7B) or in phagocytosis activity among human IgG subclasses (Figure 8B), which was assessed using an anti-human Fab capture bead and myeloma-derived human IgGs of approximately 90% purity.

**Figure 9:**
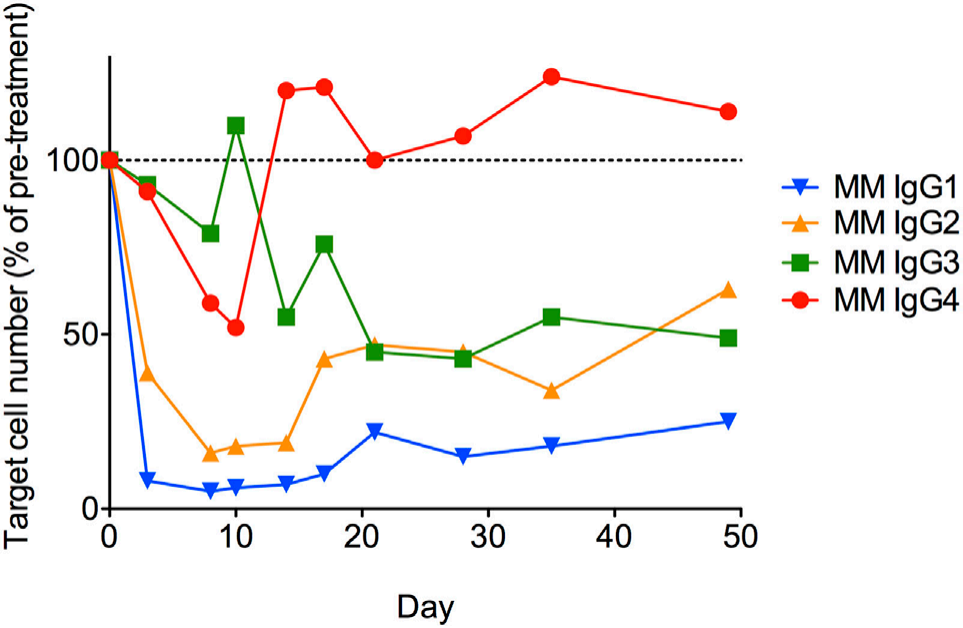
***In vivo* administration of anti-CD8α antibodies representing the four rhesus immunoglobulin G subclasses**. A single 50 mg/kg dose of each antibody was administered IV to two normal rhesus macaques, and the change from baseline in absolute number of CD3+CD4− lymphocytes in blood was assessed. Each data point represents the average (*n* = 2) percent change from baseline (average of two pretreatment measurements).

Lastly, each anti-CD8α IgG representing the four rhesus subclasses was passively administered to two rhesus macaques at 50 mg/kg, and *in vivo* effector function was assessed by quantifying CD8+ T cells in blood. To avoid cross-blocking by the administered anti-CD8α, CD8+ T cells were identified by whole blood immunophenotyping as CD3+CD4−. The percent change from baseline in the absolute number of targeted cells is shown in Figure 9. Anti-CD8α antibodies of the IgG1 and IgG2 subclass resulted in prompt loss of the targeted cell subset from blood to <20% of baseline. The mean extent and duration of CD8+ T cell depletion was greater in animals treated with IgG1 than with IgG2. The extent of CD8+ T cell depletion in animals treated with IgG3 or IgG4 never less than 40% of baseline. Furthermore, the average number of CD8+ T cells in animals treated with IgG4 exceeded the baseline values after 2 weeks. Despite the similarities in FcγR between species, the existence of several MM FcγR allotypic variants that strongly impact receptor recognition of antibody (42), or have been implicated in differential intracellular signaling (43), and the limited number of animals in this study precludes rigorous differentiation of subclass activity profiles *in vivo*. However, this depletion profile is nonetheless consistent with the biophysical and *in vitro* functional assays, suggesting that MM IgG subclasses rank by effector activity as IgG1 > IgG2 > IgG3/4. The striking differences in depletion between subclasses appear most consistent with FcγRIII-mediated activity and suggest involvement of NK cells.

## Discussion

While the effector function of polyclonal, antigen-specific antibodies elicited by candidate vaccines evaluated in NHP has often been investigated and tested *in vitro* using human effector cells (2, 5, 13, 15, 18, 44), surprisingly little is known about the immunobiology of NHP IgG subclasses and their interaction with human FcγRs. As evidence of the significance of Fc domain-driven effector functions to the *in vivo* activity of even antibodies with highly potent Fv domains continues to accumulate (3, 45–48), and the relevance that divergent subclass distributions in response to vaccination may play in efficacy in humans has become more apparent (49–51), an improved understanding of the correspondence of antibody biology between NHP and humans is likely to facilitate translational efforts centered on this model organism.

Previous studies have indicated that macaque IgG1 induced both ADCC and phagocytosis, but that the sole macaque IgG2 evaluated could not (7, 52–54). In contrast, a recent study of cynomolgus IgGs indicated that all subclasses were all able to induce lysis of opsonized B cells (11), while a previous study evaluating cynomolgus subclasses indicated otherwise (10). One confounding factor related to the existing literature regarding NHP subclasses is that some cross-species comparisons evaluate more identical sequences than within-species comparisons made across studies that have apparently utilized different allotypic variants. These allomorphs could significantly impact study results, and thus, given the limited information regarding the degree of allotypic variation among NHP subclasses, caution regarding universal application of statements as to subclass activity should be maintained. Here, we evaluated a single allotype of each MM subclass, from a widely available supply of recombinant protein, with a well-documented sequence.

We found that compared to human IgG subclasses, the MM subclasses tended to possess a more uniform binding and functional profile, with MM IgG1 generally exhibiting the highest activity, followed by IgG2, then IgG3/IgG4. Importantly, the biophysical and functional characterization here was well-supported by sequence and structural homology. Previous sequencing studies have posited that the MM IgG subclasses resulted from gene duplication events subsequent to the evolutionary branchpoint from humans based on the observation that MM subclasses are more highly related to each other than to their nominal human counterparts (7, 9). Sequence conservation among FcγR contact residues further supports the observations made here and in studies of cynomolgus macaque subclasses that IgG types may be rather more uniformly active in NHP than humans (11, 55). The biophysical features of IgG subclasses have likely evolved differently between species in part due to differences in the selective pressures individual species are exposed to in their natural environments. In particular, differences in immunological strategies addressed at bacteria, parasites, and allergens, against which humans often engender IgG2- and/or IgG4-biased responses (56–59), and even potential differences in the importance of amelioration of autoimmune or allergic responses, which are often associated with subclass switching to IgG4 in humans, between species, are suggested by these activity differences.

Notably, a series of studies have evaluated the significance of differences in IgG subclass selection on the activity and potential mechanistic link to efficacy in human HIV vaccine studies (49–51). Because rhesus macaques are the most frequently used preclinical model in HIV vaccine evaluation, there has been significant interest in understanding whether differential induction of subclasses is also associated with vaccine efficacy in macaques. However, together with evaluation of cynomolgus IgG subclasses (11), our study finds that the repertoire of antibody FcγR-binding activities present in humans is imperfectly matched to those in NHP. The more functionally inert human IgG2 and IgG4 subclasses are evidently not well mirrored among NHP subclass variants; and the functionally potentiated human IgG3 subclass bears little structural similarity to any known NHP IgG type. While human IgG1 and IgG3 demonstrate relatively similar affinities across FcγR, human IgG3 has often been observed to demonstrate superior activity in cell-based functional assays. These observations may be linked to the unique architecture of the human IgG3 hinge, which is considerably longer, bears different disulfide bonding structure, and is differentially flexible than other subclasses in humans. To the extent that these features may be mechanistically associated with the observation that IgG3 responses were potentiated among uninfected RV144 vaccine recipients (50), or factor into other candidate vaccine responses, they too may be difficult to recapitulate in NHP models.

Though the MM IgG subclasses fail to provide exact correspondence to human IgG subtypes, the relatively more uniform profile of MM IgGs likely has the advantage of potentially reducing sources of variation that could otherwise significantly impact antibody activity, the relative composition of plasma, or antigen-specific responses. Interestingly, we found in SPR experiments conducted on 10 MM plasma IgG samples that macaque serum IgG demonstrated affinity to FcγRs that were generally most similar to recombinant MM IgG1. Assuming that glycan and allotypic variance play a secondary role to subclass prevalence in FcγR-binding, this result implies that serum IgG may be predominantly comprised of the IgG1 subclass. Consistent with this possibility, but subject to limitations in detection of reagent availability and sensitivity, we observed only limited detection of IgG2 or IgG3 from two sets of MM serum IgG samples, though it is possible that allotypic variance in the IgG hinge region recognized by the anti-subclass detection reagents or other differences between naturally derived and recombinant MM IgG may also confound the ability to detect MM IgG subclasses. MS-based analysis of the subclass composition of serum IgG indicated that IgG1 was the predominant species in 18/19 animals, but levels of IgG2 estimated from standard curves accounted for an average of ~25% of serum immunoglobulin, which, based on results with mixtures of defined proportions of recombinant IgG, would have been expected to be easily detected with the subclassing reagents. Thus, while these data are not completely conclusive regarding the subclass composition of rhesus IgG, it is consistent with previous findings: a prior study found that 13 of 14 mAbs produced by rhesus heterohybridomas were consistent with IgG1, while only 1/14 was identified as an IgG2 based on restriction digests (7). In studies using RT-PCR, relative transcript expression levels were observed as follows: IgG1 > IgG2 > IgG3 > IgG4 (9); while gel-based quantitation of cDNA amplification of the hinge region indicated a relative prevalence of IgG1 >> IgG3 > IgG2 (7). Indeed, in several earlier studies, transcripts of the IgG4 subclass were not observed (7, 60).

While this study does not address potential differences in MM IgG activity due to differences in MM FcγR, our characterization of these receptors suggests that they generally exhibit similar binding preferences as human FcγR (42). However, some allotypic variants bear reduced ability to bind IgG. Additionally, some MM FcγR allotypes vary in their intracellular domains, and one of these differences was previously associated with more or less complete B cell depletion using rituximab (43). Furthermore, little is known about MM FcγR expression levels or distribution on innate immune cell subsets, which could drive dramatic differences in their activity *in vivo* as compared to human effector cell types.

Overall, we find that consistent with inferences from sequence and structural models, rhesus macaque IgG subclasses exhibit relatively uniform FcγRI and FcγRII binding and phagocytosis activity profiles—often in contrast to their nominal human IgG counterparts, which exhibit significantly greater structural and functional diversity. MM IgG1 exhibited preferential binding for human FcγRIII receptors and accordingly displayed the greatest activation of NK cells among the MM subclasses. Consistent with biophysical and cell-based assays, and suggestive of the importance of NK-mediated ADCC, passively administered anti-CD8α MM IgG1 supported the most robust depletion of CD8+ T cells *in vivo*, whereas depletion by IgG2 was moderate, and IgG3 and IgG4 demonstrated only modest (IgG3) and/or transient (IgG4) reduction.

Though previous studies have aimed to characterize the induction of specific IgG subclasses among vaccinated rhesus macaques (61, 62), collectively, the findings in this study suggest that the functional differences associated with subclass-switching profiles observed in human HIV vaccines cannot be strictly recapitulated in NHP vaccine studies. Accordingly, care must be taken not to presume the significance of “nominal” correspondence of subclass types. Finally, they also indicate that regulation of the posttranslational glycosylation of the Fc domain may play a significant role in dictating some MM IgG activities, and that glycoprofiling of vaccine-induced antibody responses could be a valuable indicator of antibody activities across species. While greater understanding of FcγR allotypes and expression patterns represent additional critical factors in developing a better understanding of the correspondence of antibody biology between species, identification of the similarities and dissimilarities between IgG subclasses is an important step to enabling both effective translation of findings in NHP studies into the clinic and the investigation of clinical correlates observed in human studies in these key model organisms.

## Ethics Statement

The study was approved by Harvard Medical School and the University of Massachusetts Medical School Institutional Animal Care and Use Committees.

## Author Contributions

AWB, NO-O, AC, TC, YC, PB, RH, AM, and AJB performed experiments and prepared figures. AWB, JW, ME Adamo, SC, and JE analyzed or interpreted data and prepared figures. AWB, SG, JS, KR, and ME Ackerman conceived and designed experiments. AWB, JS, SG, KR, and ME Ackerman wrote the manuscript. All authors read and critically reviewed the manuscript.

## Conflict of Interest Statement

The authors declare that the research was conducted in the absence of any commercial or financial relationships that could be construed as a potential conflict of interest.

## Footnotes

^1^http://www.ncbi.nlm.nih.gov/protein/XP_001096027.2.

^2^http://www.ncbi.nlm.nih.gov/protein/XP_001106090.1.
