# Supplementary material for: Biophysical and Functional Characterization of Rhesus Macaque IgG Subclasses
Source: Front Immunol. 2016 Dec 13;7:589. doi: 10.3389/fimmu.2016.00589 (PMC5153528; doi:10.3389/fimmu.2016.00589)
Supplement: Supplementary file 2 [file presentation_1.pptx]

## Slide 1
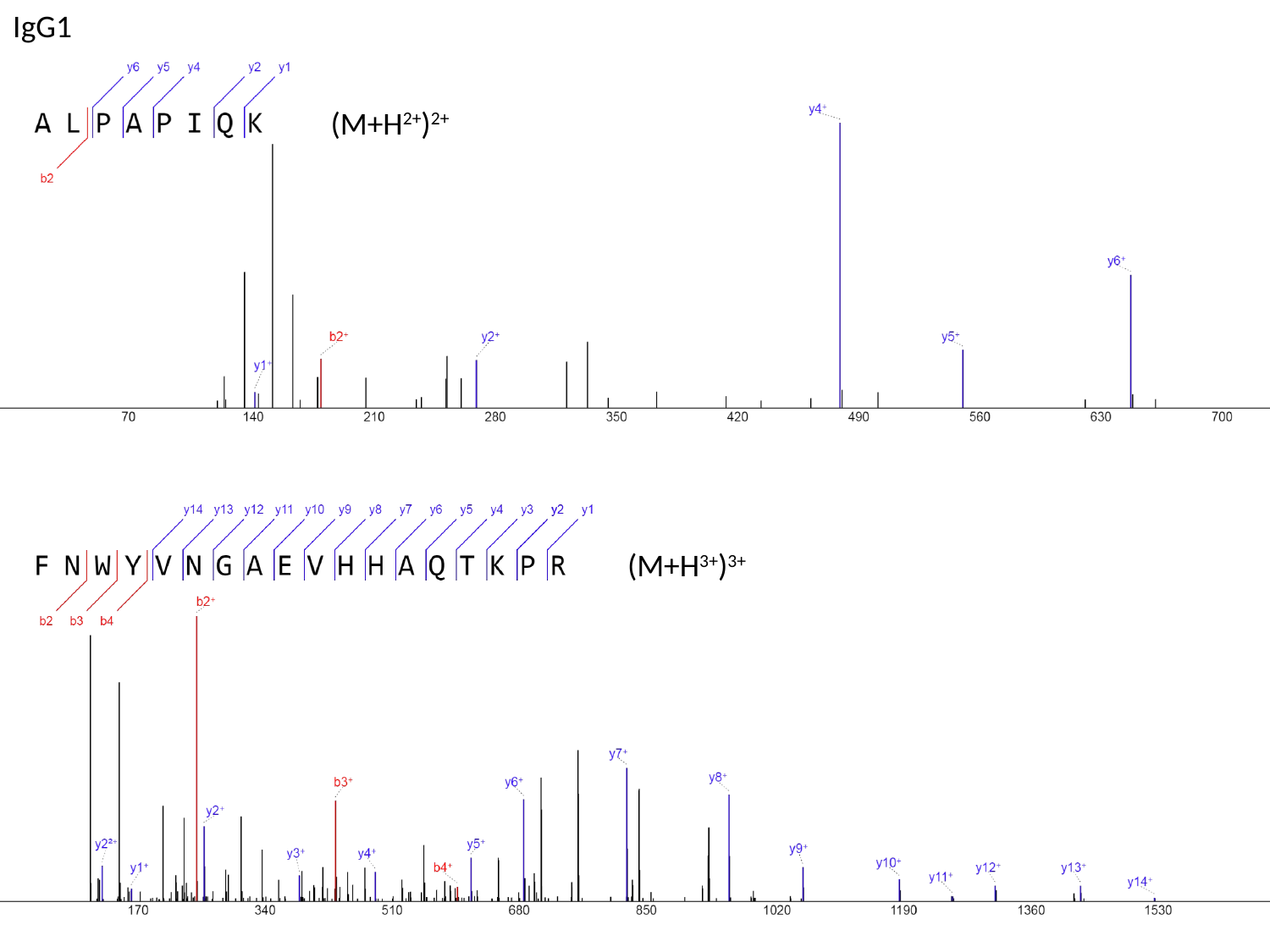

IgG1
(M+H2+)2+
(M+H3+)3+

## Slide 2
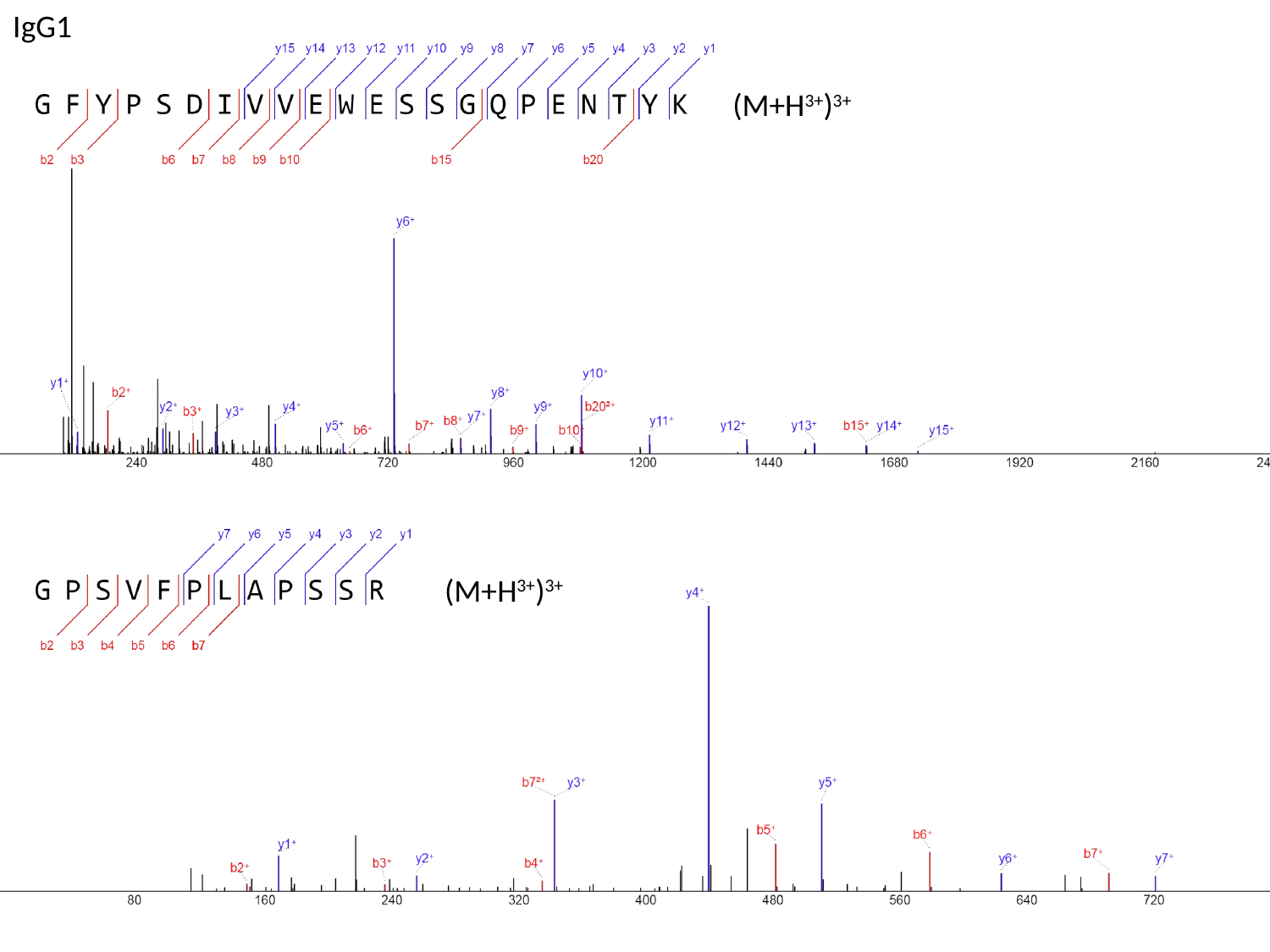

IgG1
(M+H3+)3+
(M+H3+)3+

## Slide 3
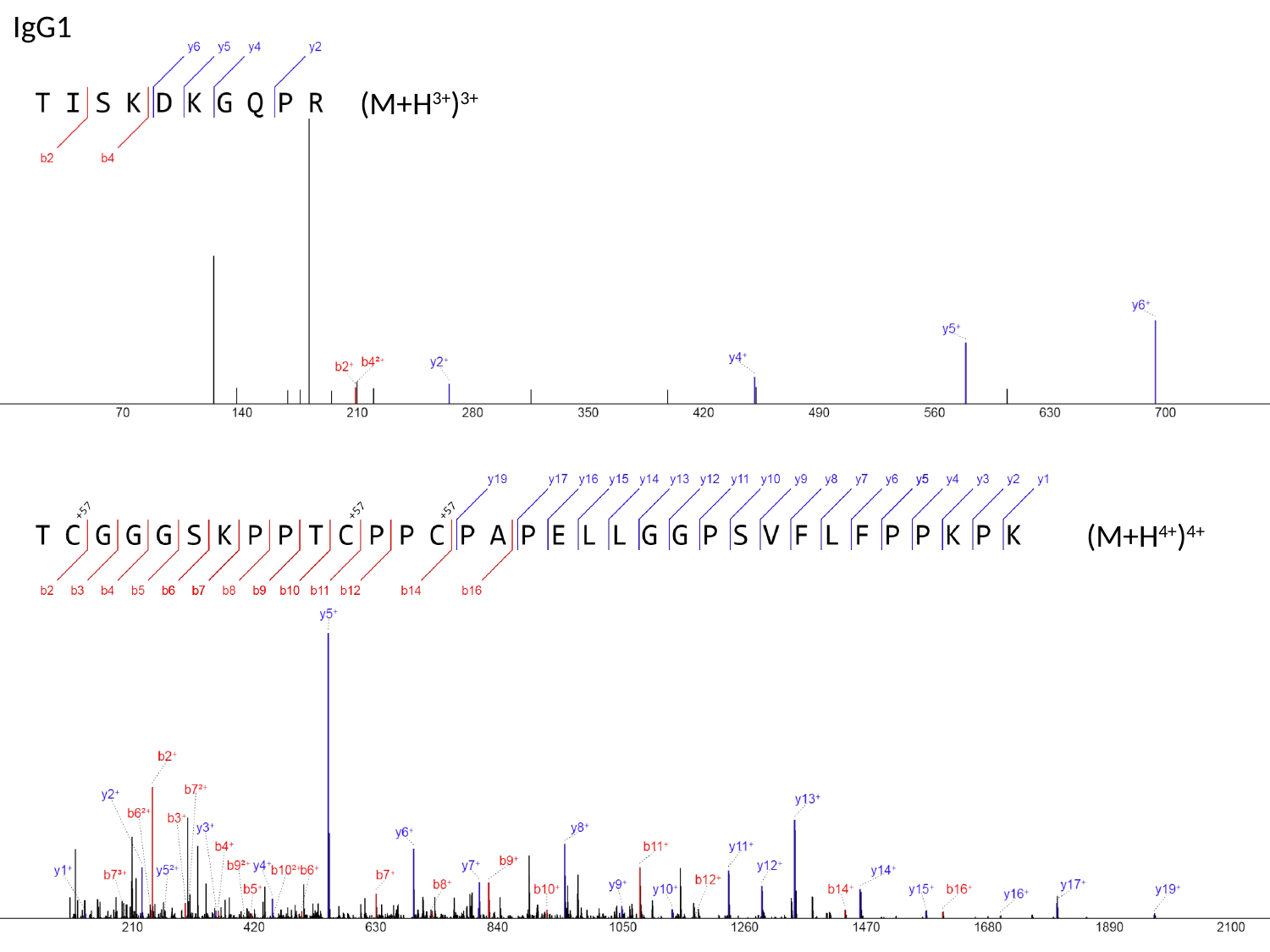

IgG1
(M+H3+)3+
(M+H4+)4+

## Slide 4
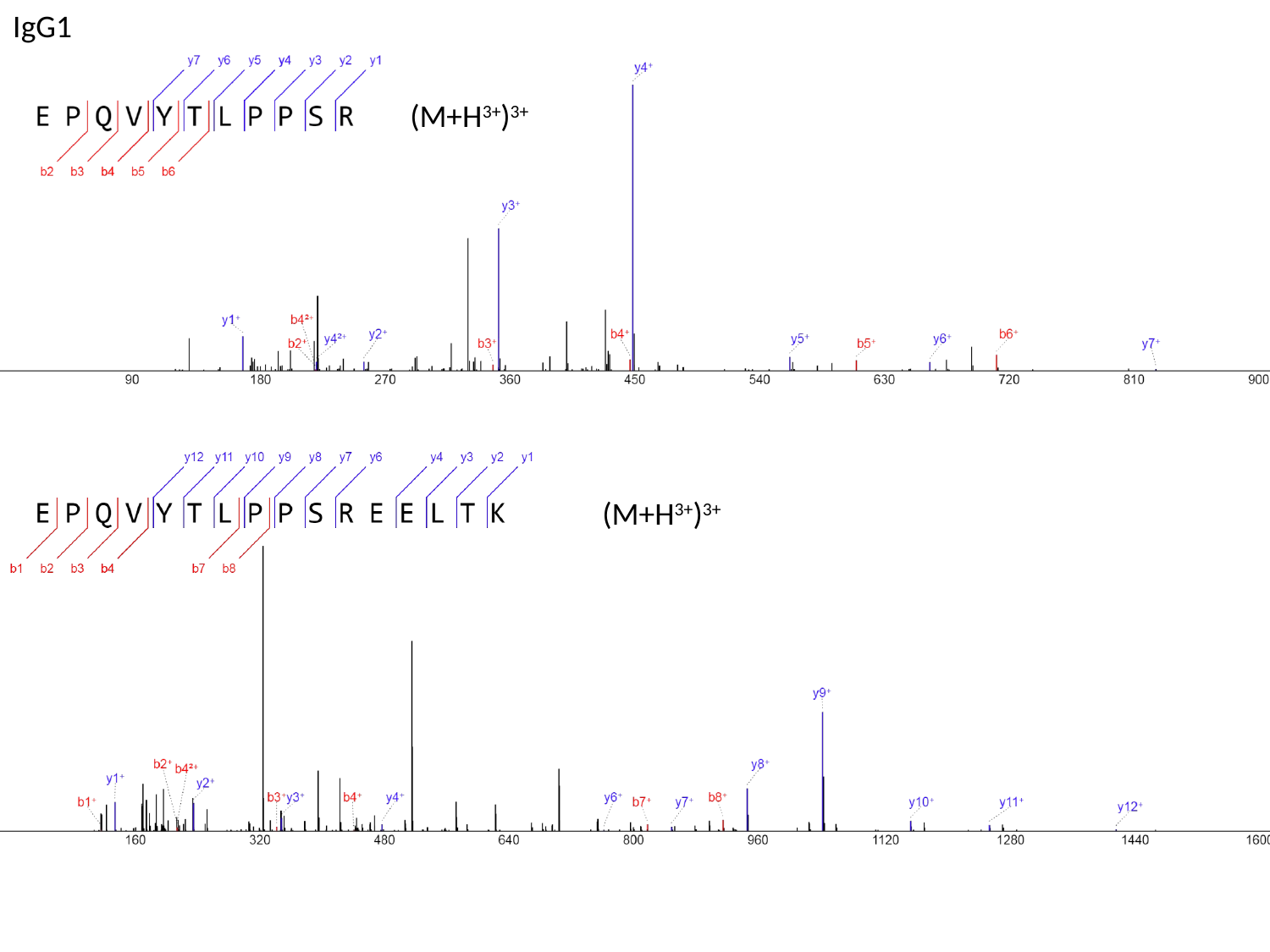

IgG1
(M+H3+)3+
(M+H3+)3+

## Slide 5
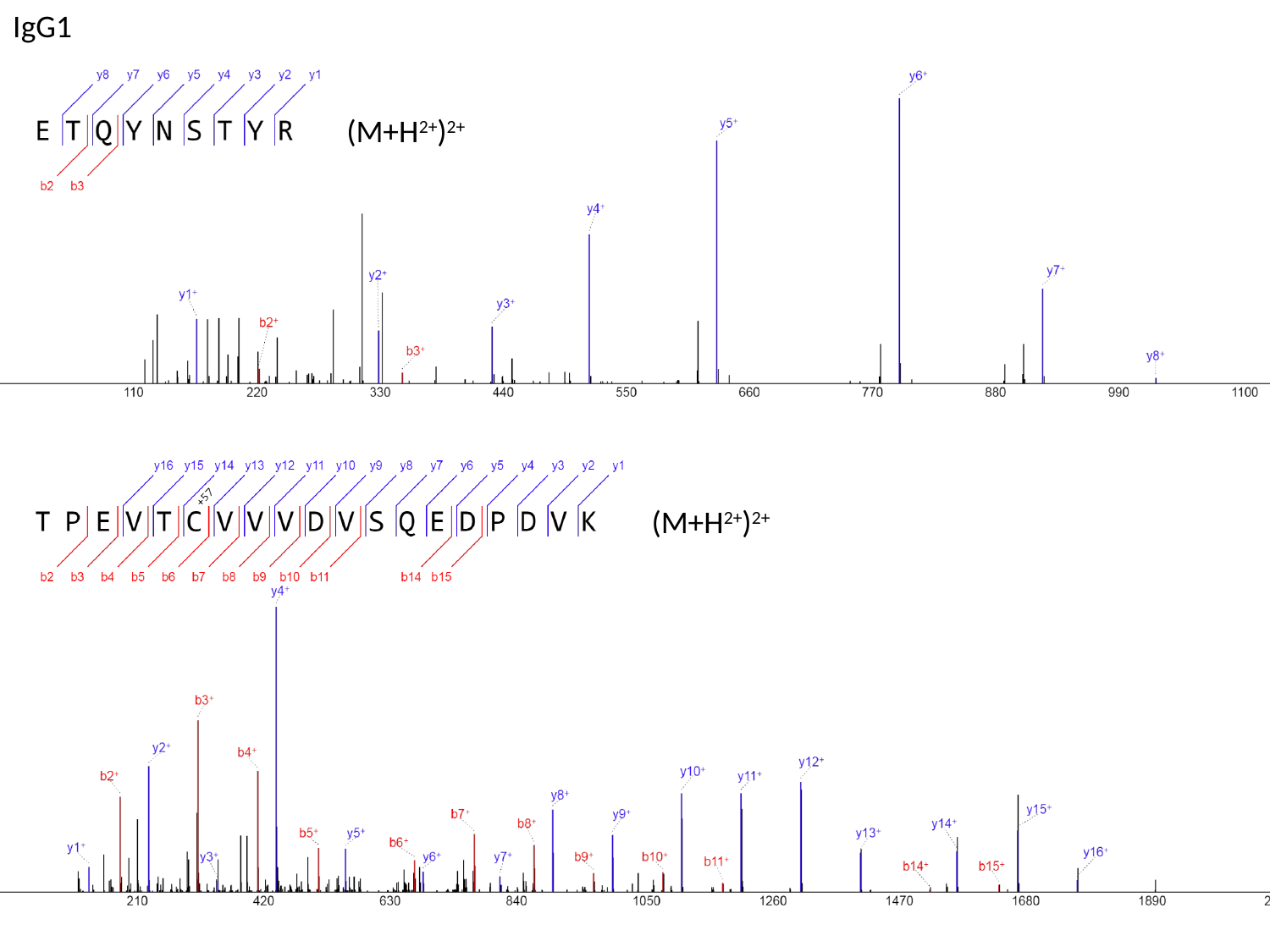

IgG1
(M+H2+)2+
(M+H2+)2+

## Slide 6
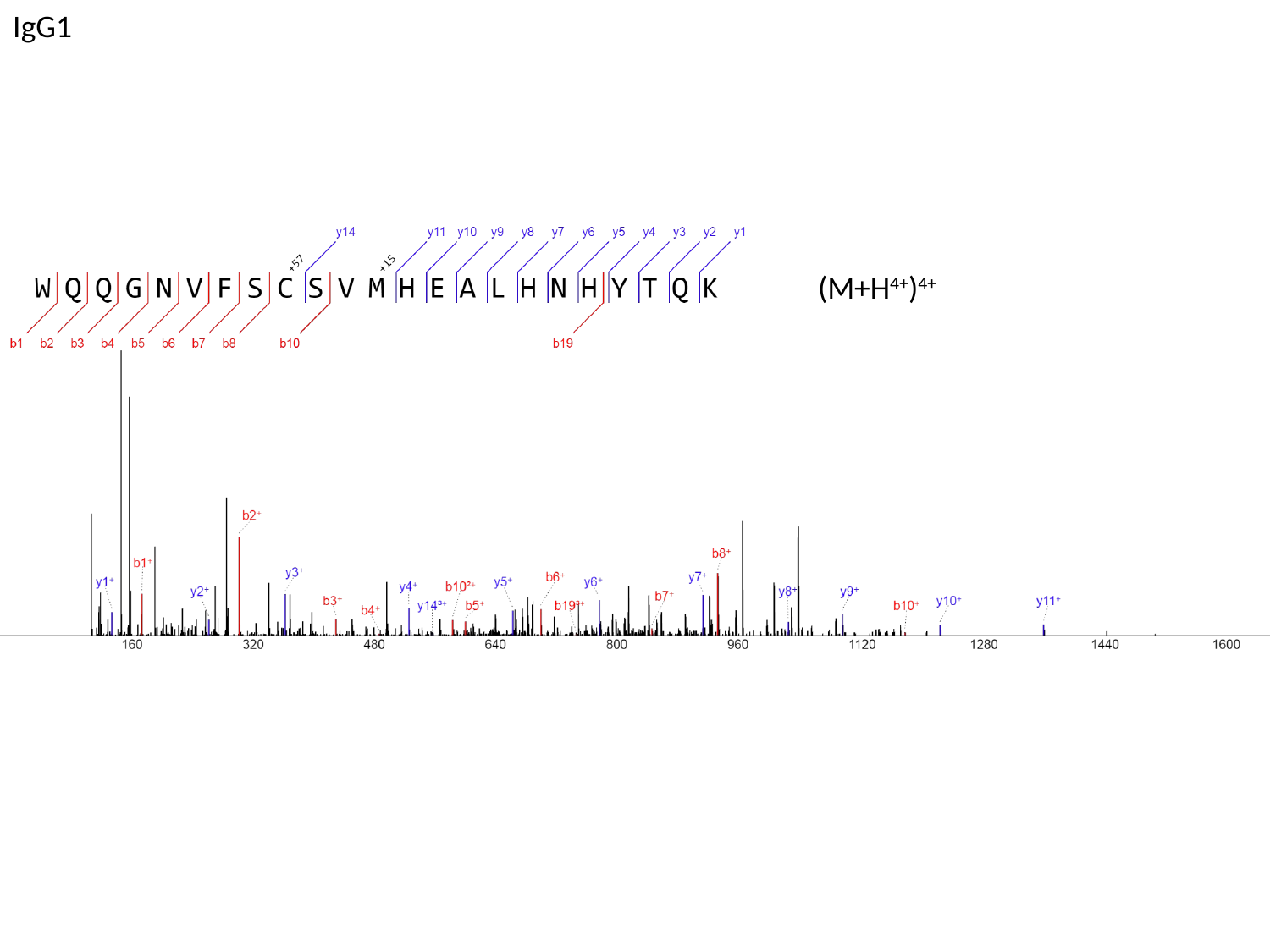

IgG1
(M+H4+)4+

## Slide 7
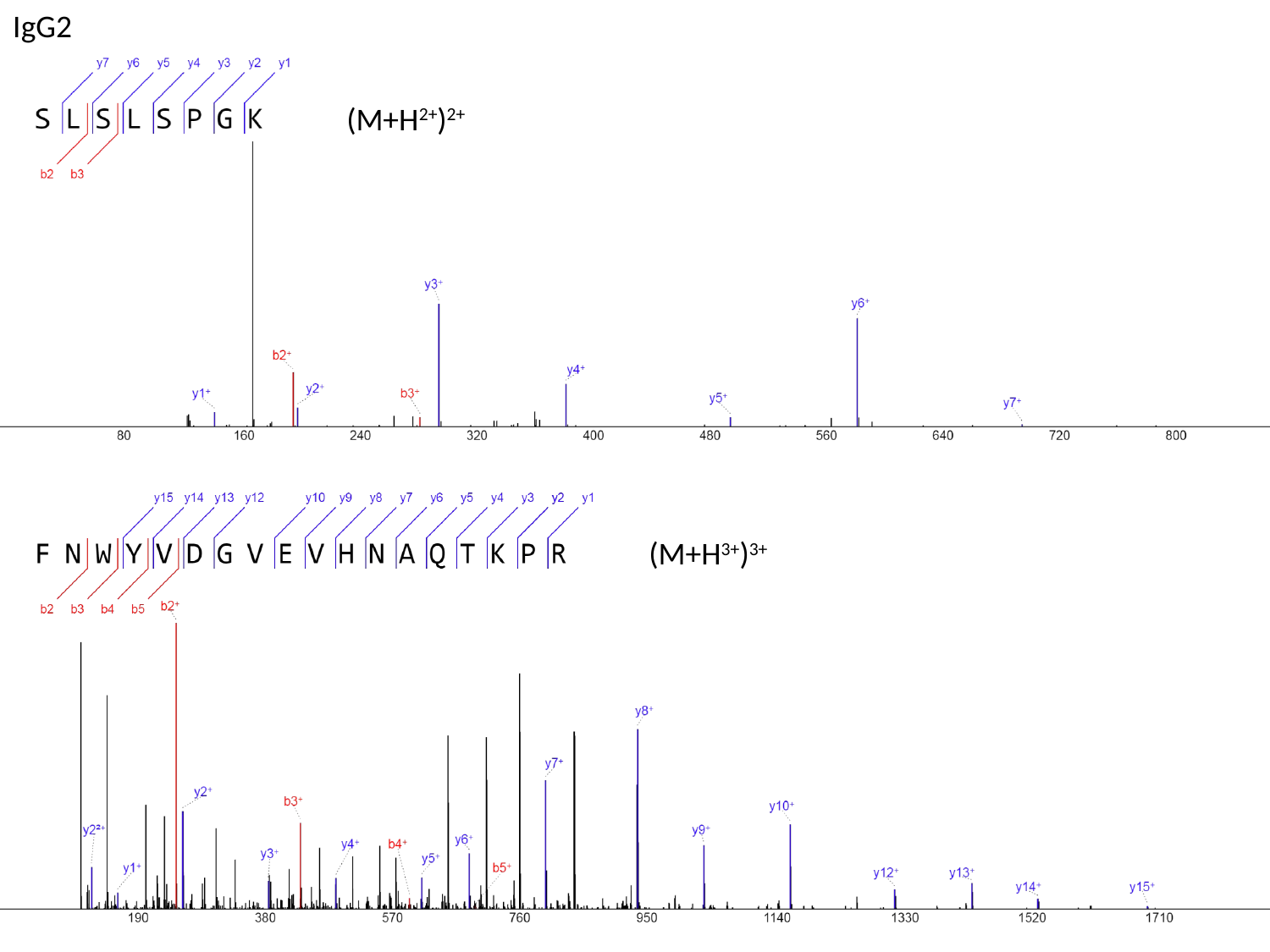

IgG2
(M+H2+)2+
(M+H3+)3+

## Slide 8
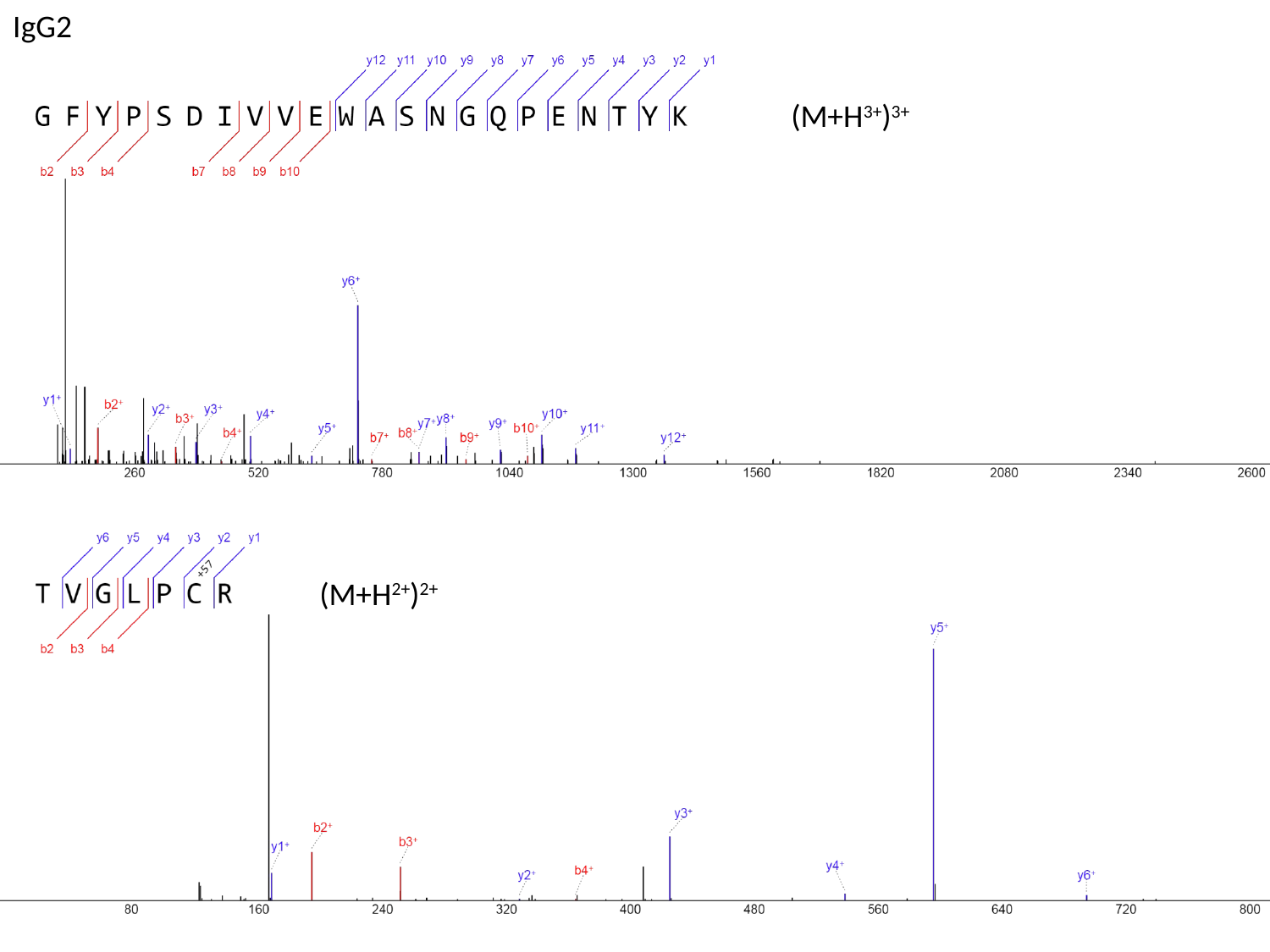

IgG2
(M+H3+)3+
(M+H2+)2+

## Slide 9
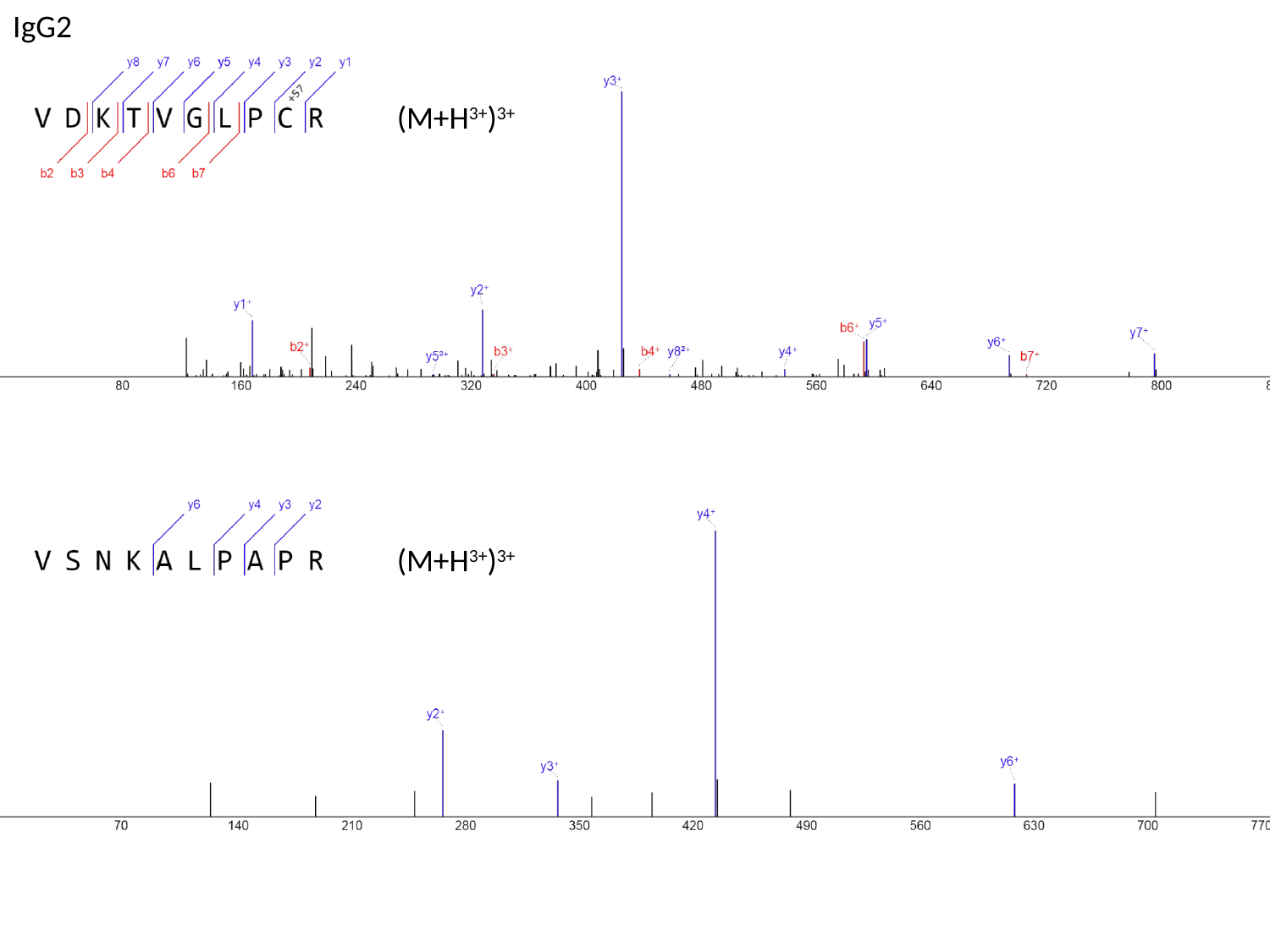

IgG2
(M+H3+)3+
(M+H3+)3+

## Slide 10
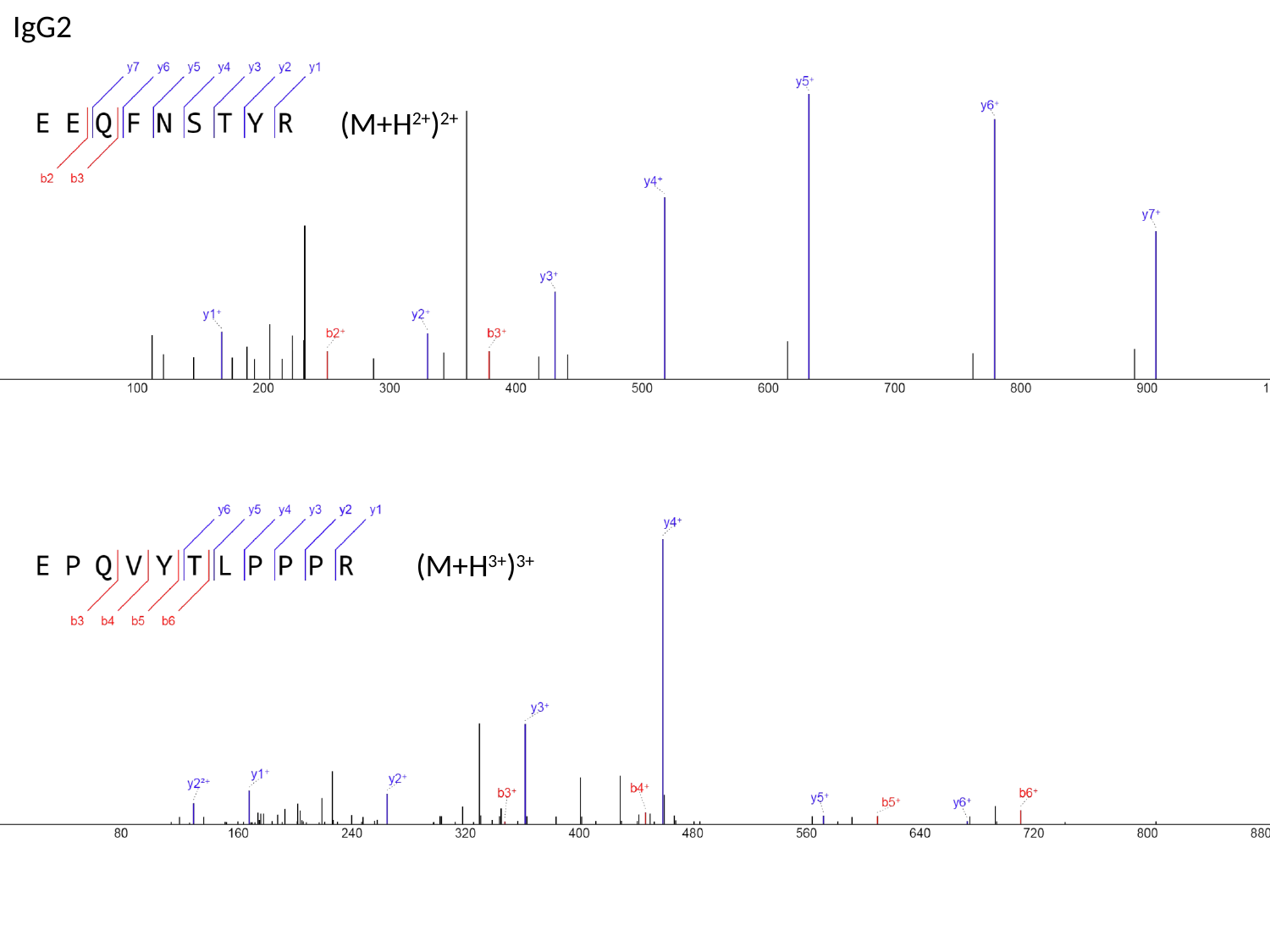

IgG2
(M+H2+)2+
(M+H3+)3+

## Slide 11
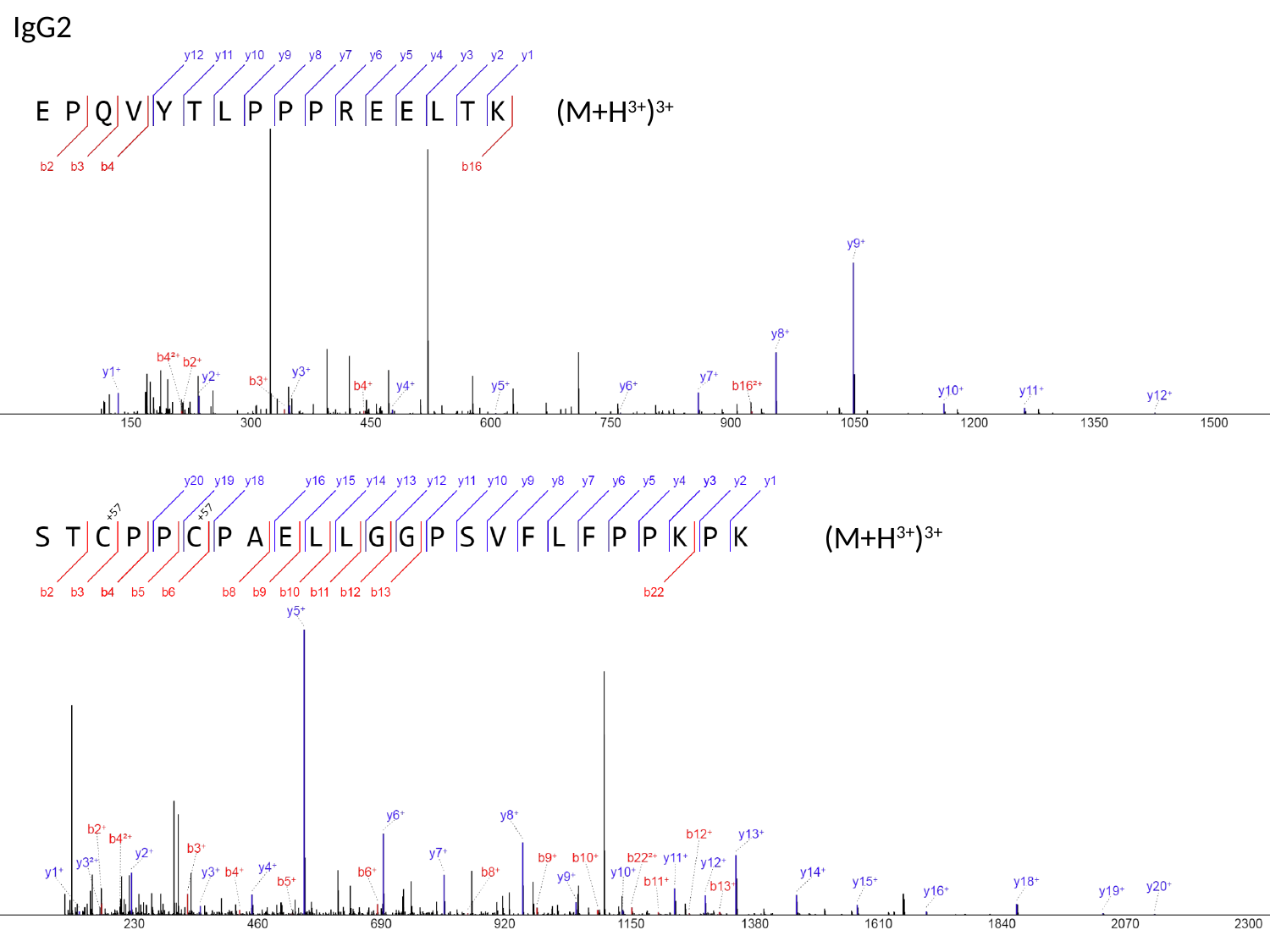

IgG2
(M+H3+)3+
(M+H3+)3+

## Slide 12
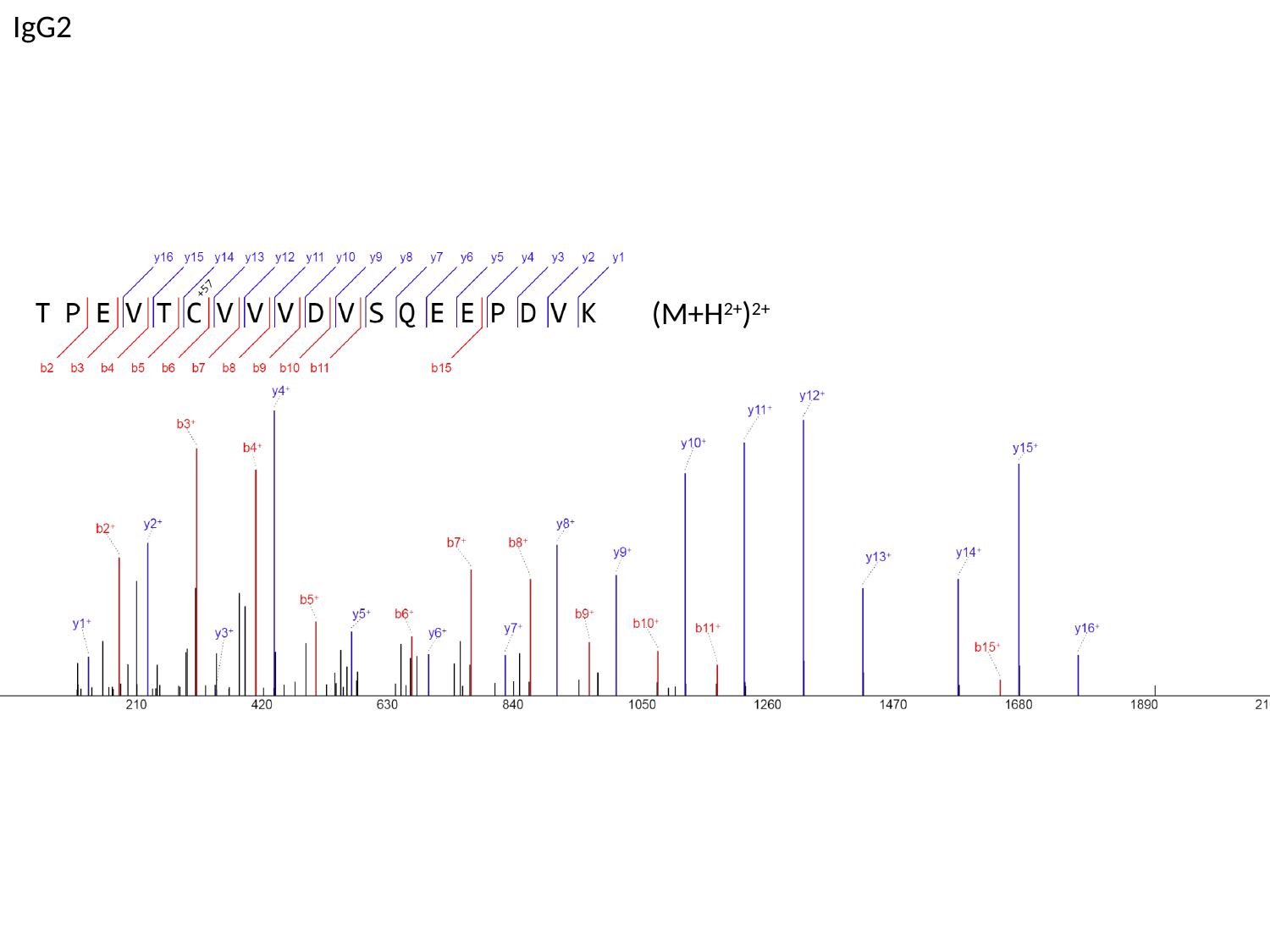

IgG2
(M+H2+)2+

## Slide 13
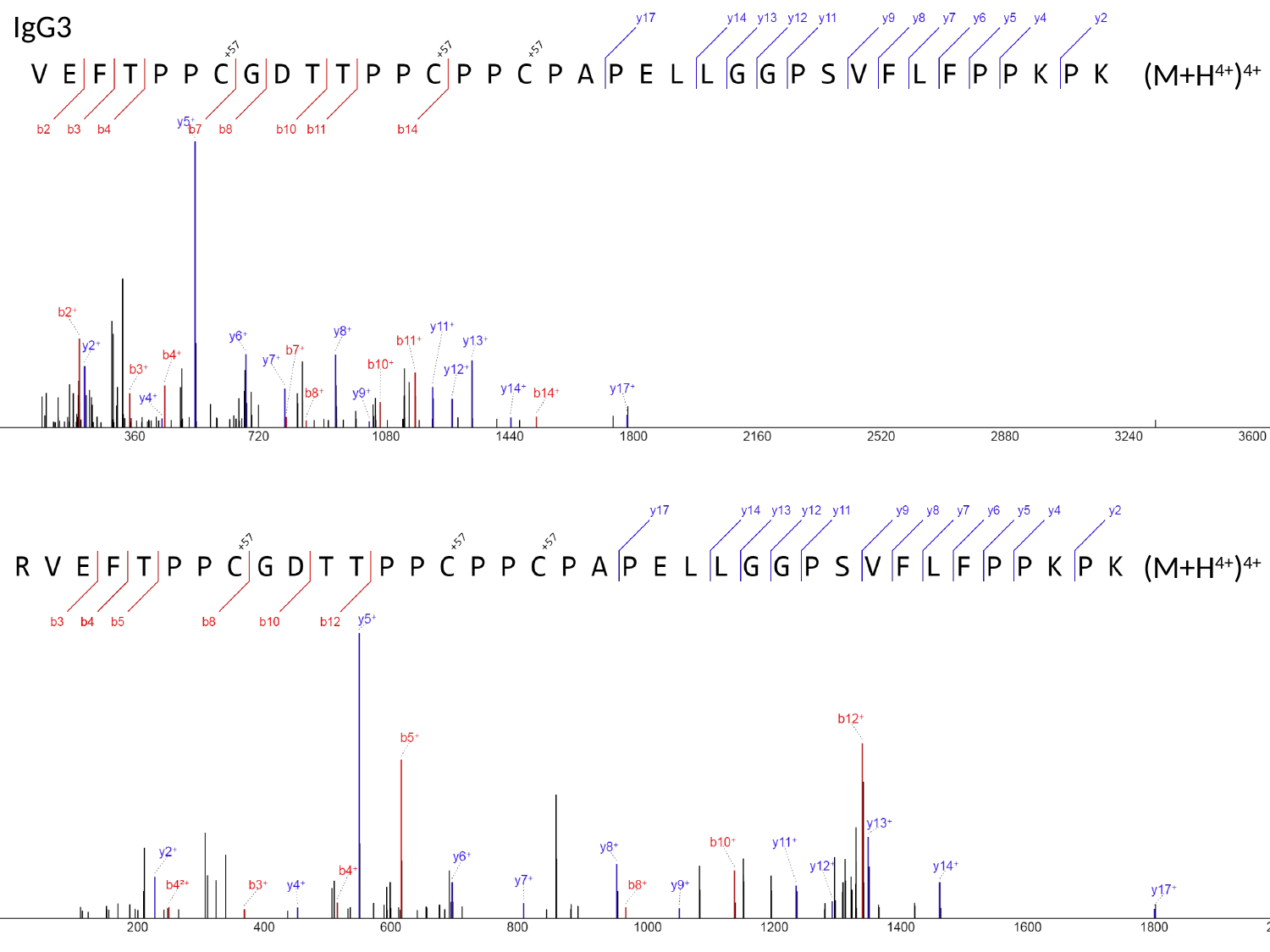

IgG3
(M+H4+)4+
(M+H4+)4+

## Slide 14
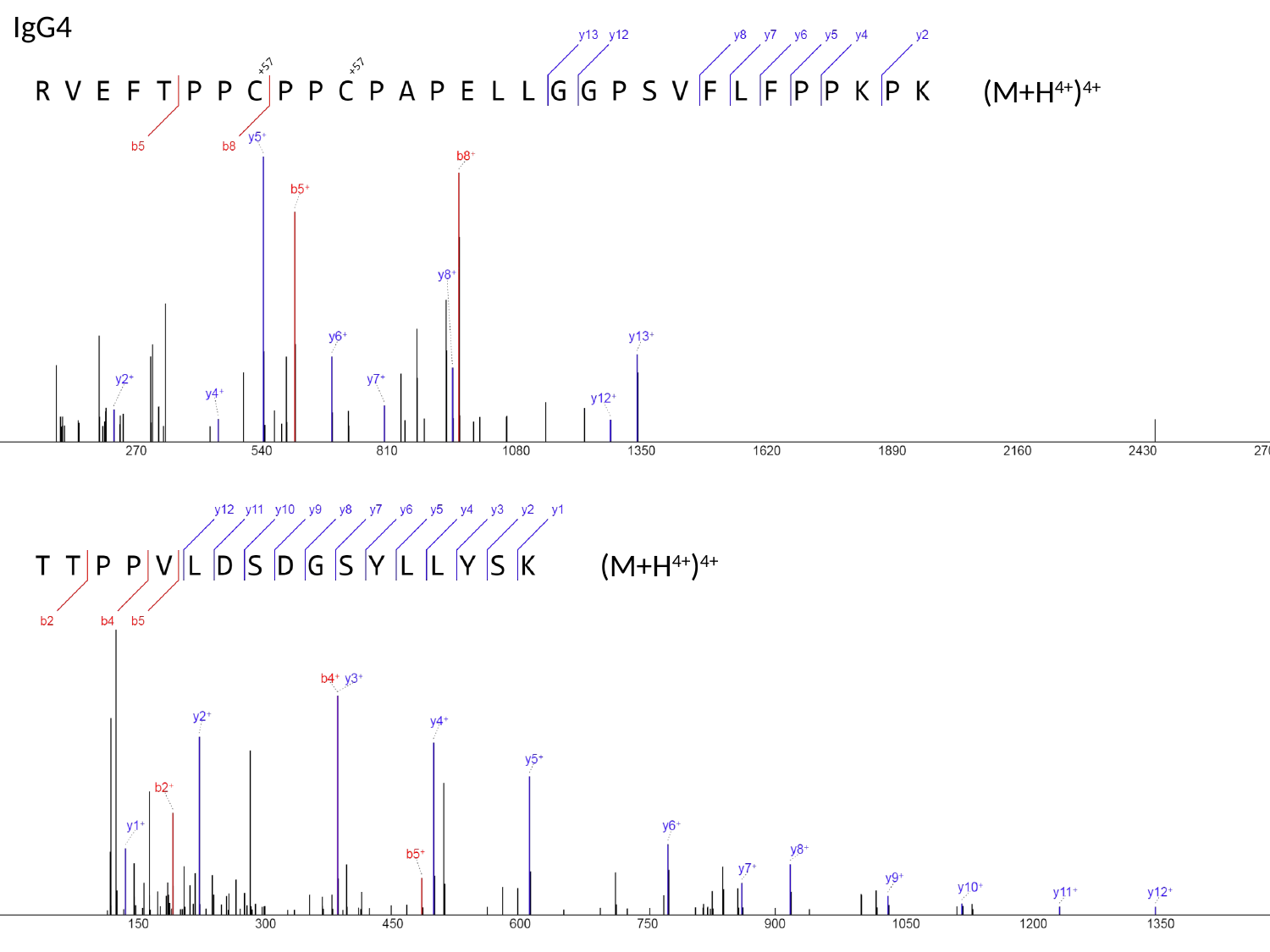

IgG4
(M+H4+)4+
(M+H4+)4+

## Slide 15
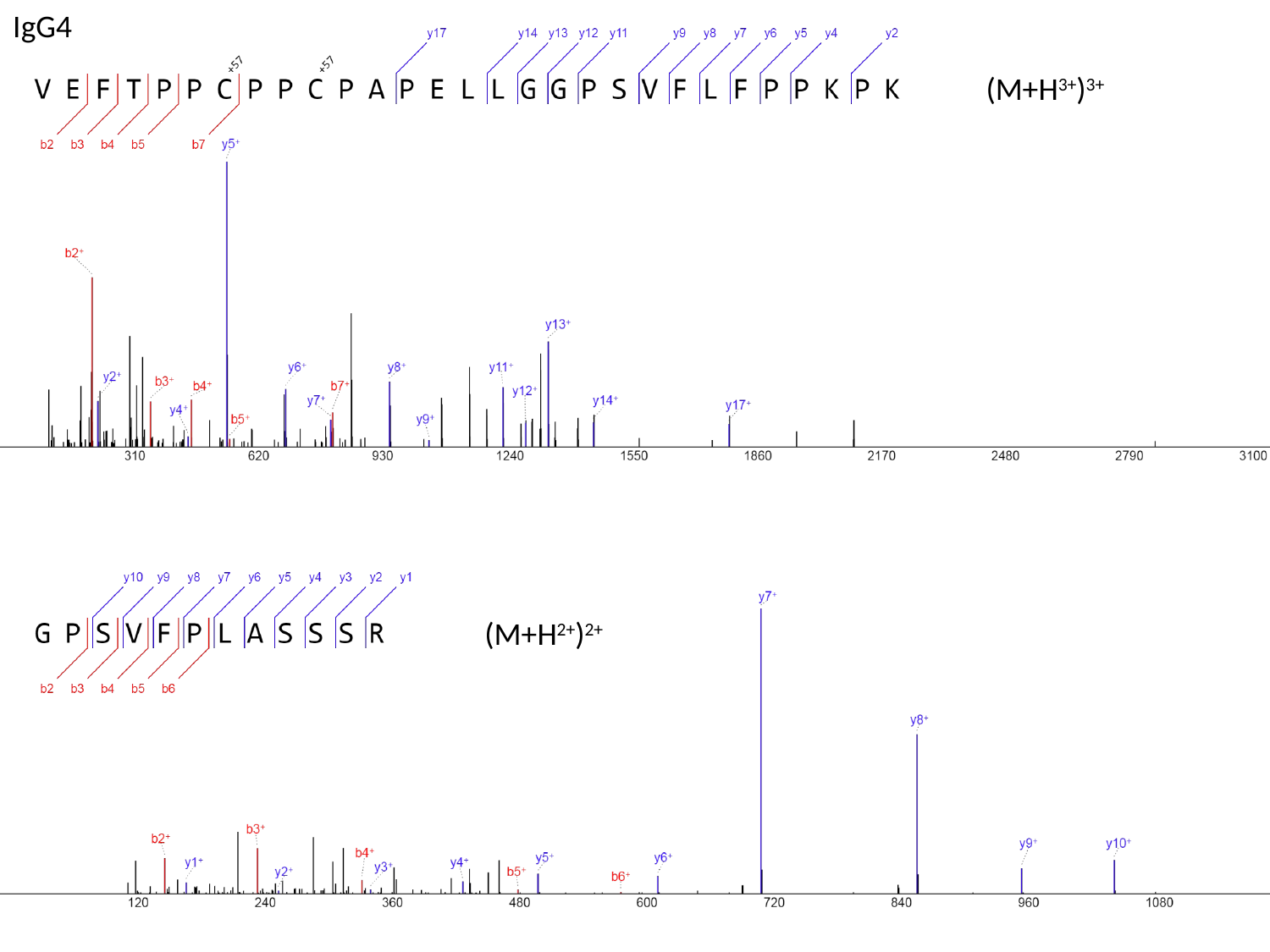

IgG4
(M+H3+)3+
(M+H2+)2+

## Slide 16
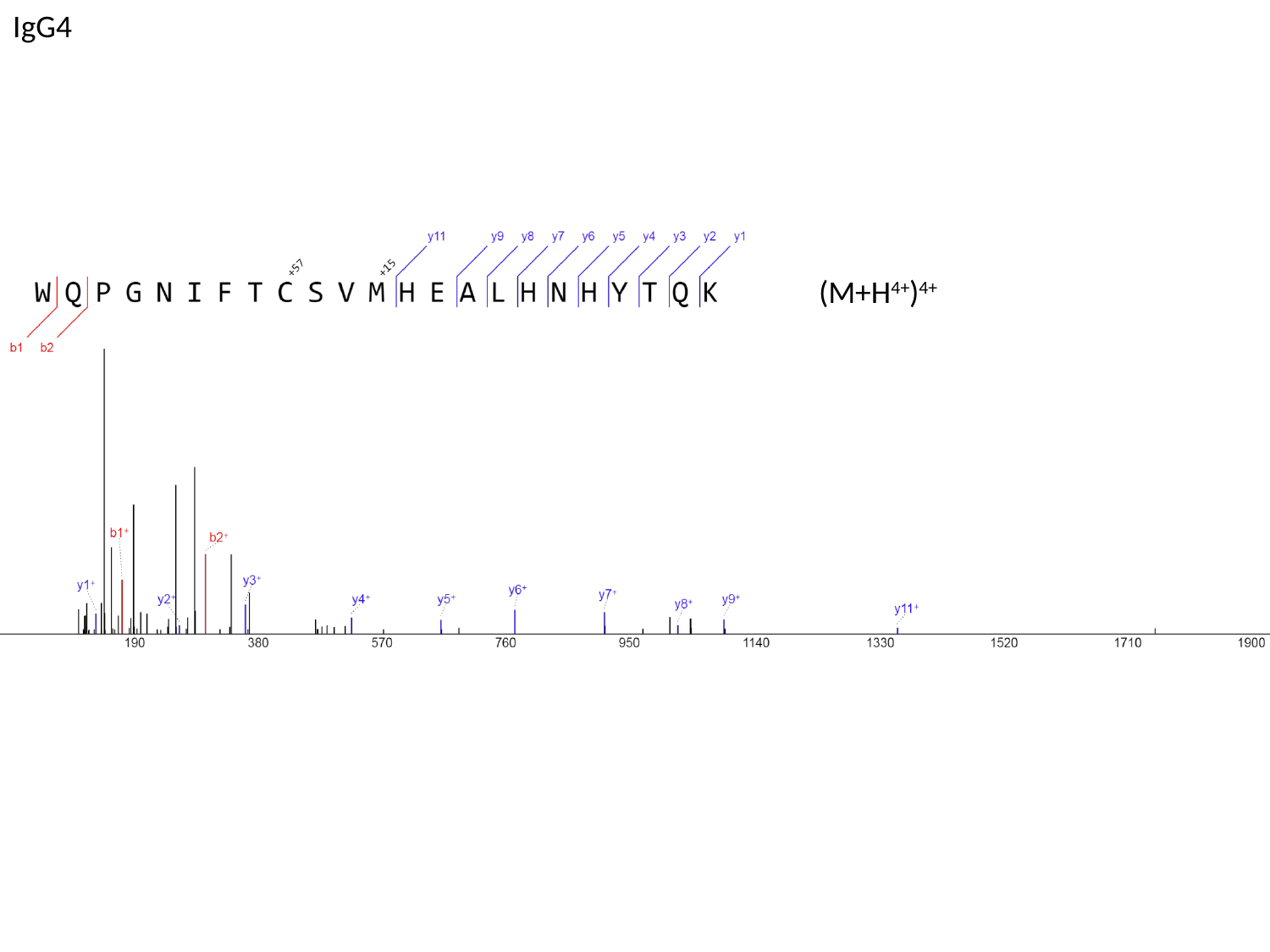

IgG4
(M+H4+)4+
